# Evidence for trans-synaptic propagation of oligomeric tau in human progressive supranuclear palsy

**DOI:** 10.1038/s41593-025-01992-5

**Published:** 2025-07-16

**Authors:** Robert I. McGeachan, Lois Keavey, Elizabeth M. Simzer, Ya Yin Chang, Jamie L. Rose, Maxwell P. Spires-Jones, Mollie Gilmore, Kristjan Holt, Soraya Meftah, Natalia Ravingerova, Cristina Scutariu, Lewis W. Taylor, Declan King, Makis Tzioras, Jane Tulloch, Sam A. Booker, Imran Liaquat, Nicole Hindley-Pollock, Bethany Geary, Colin Smith, Paul M. Brennan, Claire S. Durrant, Tara L. Spires-Jones

**Affiliations:** 1 The University of Edinburgh Centre for Discovery Brain Sciences, Edinburgh, UK; 2 UK Dementia Research Institute at The University of Edinburgh, Edinburgh, UK; 3 The University of Edinburgh Hospital for Small Animals, Edinburgh, UK; 4 Scottish Brain Sciences, Edinburgh, UK; 5 The University of Edinburgh Centre for Clinical Brain Sciences, Edinburgh, UK; 6 MRC Protein Phosphorylation and Ubiquitylation Unit, University of Dundee, Dundee, UK

**Keywords:** Neurodegeneration, Neurological disorders

## Abstract

In the neurodegenerative disease progressive supranuclear palsy (PSP), tau pathology progresses through the brain in a stereotypical spatiotemporal pattern, and where tau pathology appears, synapses are lost. We tested the hypothesis that pathological tau contributes to synapse loss and may spread through the brain by moving from presynapses to postsynapses. Using postmortem PSP brain samples and a living human brain slice culture model, we observe pathological tau in synaptic pairs and evidence that oligomeric tau can enter live human postsynapses. Proteomics revealed increased clusterin in synapses in PSP, and super-resolution imaging showed clusterin colocalized with tau in synapses in close enough proximity to be binding partners, which may mediate tau spread. Accumulation of tau in synapses correlated with synapse loss, and synaptic engulfment by astrocytes was observed, suggesting that astrocytes contribute to synapse loss. Together, these data indicate that targeting synaptic tau is a promising approach to treat PSP.

## Abstract (web summary)

McGeachan et al. observe oligomeric tau in synapses from individuals with progressive supranuclear palsy and provide evidence that tau pathology may spread through the brain via synapses.

## Main

Progressive supranuclear palsy (PSP) is a neurodegenerative disease characterized by accumulation of tau pathology in neurons, oligodendrocytes and astrocytes, accompanied by impairments in movement, balance, cognition and behavior^1^. Tau pathology accumulates in the brain in a stereotypical spatiotemporal pattern as PSP progresses^2^. Areas with a high tau burden show synaptic loss, and the degree of synapse loss over time correlates with the progression of disease symptoms^3,4^. Tau protein can bind synaptic vesicles and affect synaptic function^5^, and pathological phosphorylation and aggregation of tau cause synaptic dysfunction and loss in model systems^6–8^. Therefore, understanding the mechanisms of tau propagation and how pathological tau causes synapse loss is important for future therapeutic development to slow or stop symptoms of PSP.

The mechanisms driving tau propagation and synaptic loss in PSP remain largely unknown. Although one hypothesis is that the stereotypical tau accumulation in sequential brain regions in tauopathies is due to different regions being differentially vulnerable to local pathological changes in tau^9^, there is strong evidence suggesting that pathologically aggregated and phosphorylated tau spreads physically through the brain both within local regions and between different brain regions^10^. In mouse models of tauopathy, tau pathology can spread between brain regions by being released from presynaptic terminals and taken up by connected postsynapses. For example, in mice expressing mutant human tau with expression restricted to neurons in the entorhinal cortex, tau pathology spreads to connected dentate gyrus neurons (which do not express human tau) via presynapse to postsynapse spread^11–16^. More specific to PSP, injecting extracts of postmortem PSP brain into mice expressing wild-type human tau induces the formation of neuronal and glial inclusions that spread through the brain^17^, and recent in vivo magnetic resonance imaging research suggests that the progression of tau pathology in human PSP occurs along functionally connected brain regions^18^.

Recently, our group used array tomography and electron microscopy to study synaptic accumulation and potential trans-synaptic propagation of tau in Alzheimer’s disease (AD), the most common tauopathy. We observed that oligomeric tau accumulates in synaptic pairs, supporting the potential spread between synaptically connected neurons^10^. Furthermore, oligomeric tau was observed in presynaptic terminals in the occipital cortex, one of the last brain areas in AD to accumulate tau pathology. This supports the hypothesis that presynaptic release of oligomers from affected brain regions is a mechanism of pathological tau spread^10^. Although fibrillar tau deposits, such as neurofibrillary tangles (NFTs) and tufted astrocytes, are the pathological hallmarks of tauopathies, research in model systems suggests that these are likely end-stage aggregates and do not themselves induce neurodegeneration or functional abnormalities^19,20^. There is a growing body of evidence that suggests that soluble tau oligomers are the source of toxicity, instead of large pathological aggregates, including NFTs. Soluble tau oligomers induce early synaptic dysfunction, synaptic and neuronal degeneration and have a greater propensity for propagation and seeding than fibrils^21–23^. One potential mechanism of synaptotoxicity is through oligomeric tau binding to synaptic vesicles and impairing neurotransmitter release. In postmortem tissue from individuals with AD and in *Drosophila* and mouse models, tau colocalizes with the presynaptic vesicle protein synaptogyrin-3 and lowering synaptogyrin-3 levels prevented synapse loss and memory decline^7,8,24^.

Another potential mechanism leading from pathological tau to synapse loss is via aberrant induction of synaptic pruning by glia. Synaptic pruning mediated by astrocytes and microglia is an important aspect of normal circuit development^25,26^. Recently, we observed astrocytes and microglia engulf synapses in AD, with astrocytes containing more synaptic proteins than microglia^27^. Furthermore, in AD, presynapses containing p-tau Ser356 are five times more likely to colocalize with astrocytes^28^. In addition, in postmortem Alzheimer’s brain, synapses colocalizing with oligomeric tau are more likely to colocalize with microglia and astrocytes^29^. This suggests that the presence of tau oligomers in synapses may serve as signals for increased glial-mediated synapse engulfment in AD. In AD, amyloid pathology is thought to drive a large amount of the observed neuroinflammation. We observed that glial engulfment of synapses was greatest in the vicinity of amyloid plaques^27^. Less is known about glial engulfment of synapses in primary tauopathies like PSP despite prominent astrocyte pathology.

While synapse loss and the spread of tau pathology have been observed in PSP as detailed above, there have not previously been detailed investigations of synaptic tau or potential mechanisms of tau spread and toxicity in human PSP. Using postmortem PSP brain and living human brain slice cultures (HBSCs), here we test the hypothesis that tau pathology could spread trans-synaptically in PSP and that synaptic accumulation of tau may contribute to synapse loss by inducing molecular changes in synapses and synaptic engulfment by astrocytes.

## Results

### Phosphorylated tau colocalizes with synaptic vesicle proteins in PSP

**Fig. 1:**
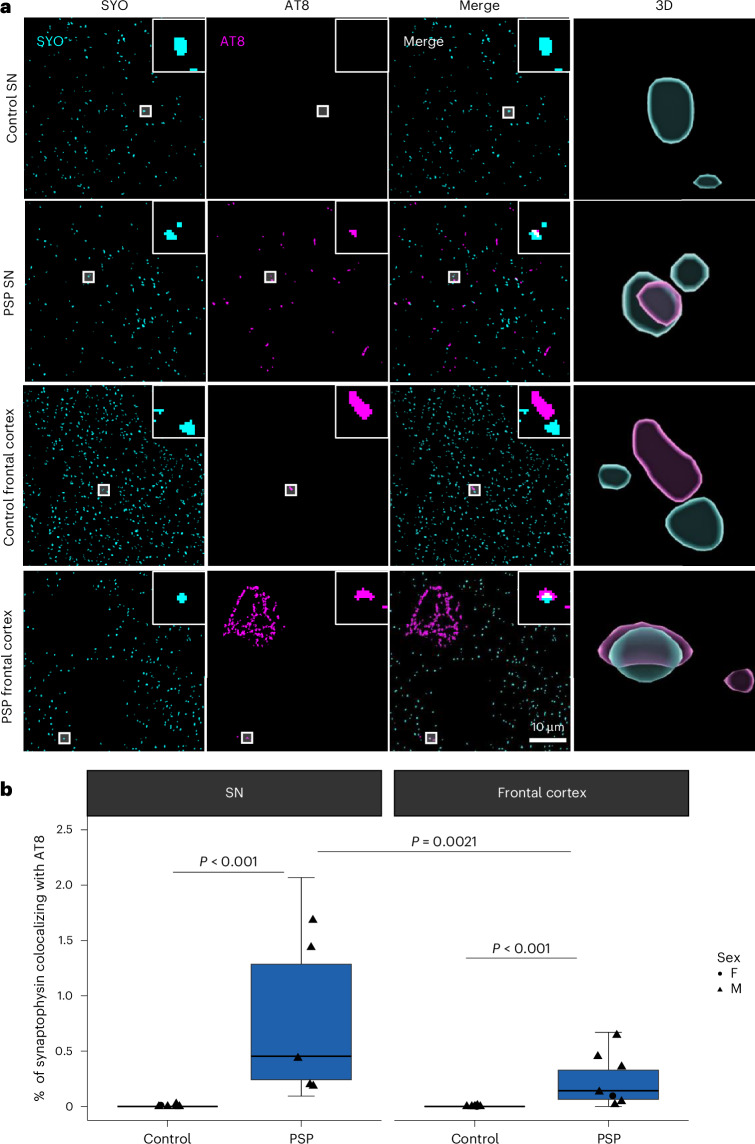
Phospho-tau accumulates within presynaptic terminals in PSP. **a**, Array tomography ribbons were immunostained for SYO (cyan) and phospho-tau Thr202, Ser205 (AT8, magenta). Single 70 nm segmented sections show representative staining in the SN and frontal cortex of control and PSP brains. Insets highlight colocalization between SYO and AT8. 3D reconstructions made using IMARIS. Scale bar = 10 μm. Large boxes = 50 μm × 50 μm. Insets = 2 μm × 2 μm. **b**, Quantification of colocalization shows an increase in the percentage of SYO puncta colocalizing with AT8 in PSP brain (*n* = 5 donors SN, 7 frontal cortex) compared to control (*n* = 6 SN, 7 frontal cortex). Within PSP brain, there are more SYO objects colocalizing with AT8 in the SN compared to the frontal cortex. Boxplots show the first quartile to third quartile (box) with the median marked, and whiskers show maximum and minimum values calculated from each image stack, excluding outliers (two image stacks per region per case were taken as technical replicates). Data points show case means (biological replicates). Data were Tukey transformed and analyzed with LMEM (~diagnosis × brain region + 1 | case, ANOVA after LMEM effect of disease *F*(1,11.58) = 74.587, *P* < 0.0001) with post hoc pairwise Tukey-corrected *P* values showing significant effect of disease in both SN (estimate = 0.760, 95% CI (0.561, 0.959), *t*(23.8) = 7.887, *P* < 0.0001) and frontal cortex (estimate = 0.557, 95% CI (0.377, 0.738), *t*(19.5) = 5.918, *P* < 0.0001). Within PSP cases, the proportion of AT8-positive synapses is higher in the SN than the frontal cortex (estimate = 0.242, 95% CI (0.0932, 0.390), *t*(25.3) = 3.293, *P* = 0.0021). F, female; M, male.

**Extended Data Fig. 1:**
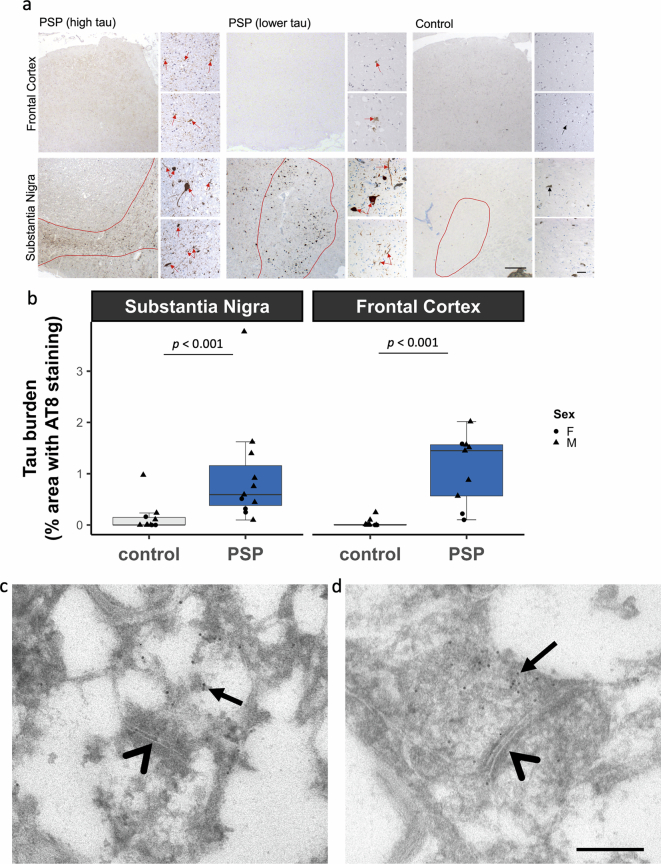
Increased phospho-tau Ser202, Thr205 in PSP substantia nigra and frontal cortex and validation of synaptic staining. **a**, Representative images of cases with high and low tau burdens of formalin-fixed, paraffin-embedded slides from PSP and control frontal cortex and substantia nigra immunolabeled for phospho-tau Ser202, Thr205 with the AT8 antibody. Large box scale bar = 500 µm, Small box scale bar = 50 µm. Red arrows highlight tau/DAB staining. Black arrows highlight artifact or pigments that is not real tau/DAB staining. **b**, Quantification of AT8 staining identifies an increase in phospho-tau Ser202, Thr205 in PSP substantia nigra and frontal cortex compared to age and sex matched controls. Boxes show quartiles and medians calculated from each image; whiskers show maxima and minima excluding outliers. Data points show case means (females, circles; males, triangles, n = 9 PSP, 9 control cases frontal cortex, n = 10 control, 11 PSP cases substantia nigra, one slide per case per region imaged). Data were analyzed with linear mixed effects models (tau_burden ~ diagnosis × brain_region + 1 | case). *P* values were calculated from post hoc testing with Tukey correction for multiple comparisons. AT8 tau burden is higher in PSP substantia nigra (estimate = 0.642, 95% CI (0.36,0.924), *t*(32.1) = 4.638, *p* = 0.0001) and in frontal cortex (estimate = 0.806, 95% CI (0.506,1.107), *t*(33.7) = 5.453, *p* < 0.0001). **c**,**d**, Validation with immunogold electron microscopy that synaptophysin antibody labels presynaptic vesicles (10 nm gold-conjugated to secondary antibody indicated with arrows), which are opposed to electron dense post-synaptic densities (arrowheads) in both postmortem PSP brain tissue (**c**) and human brain slices (**d**). Scale bar = 200 nm.

**Extended Data Fig. 2:**
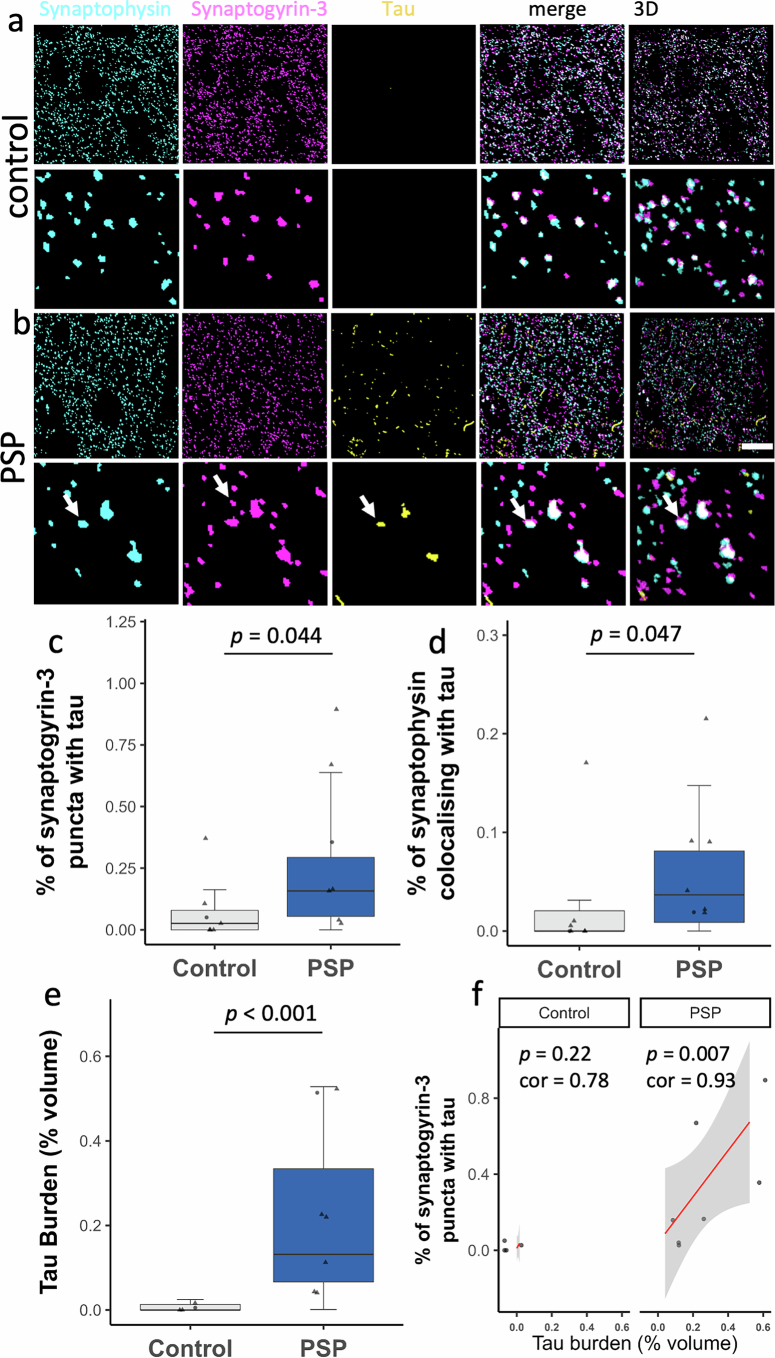
Synaptic tau colocalises with synaptogyrin-3 in PSP frontal cortex. **a**,**b**, Segmented array tomography images. Array tomography ribbons were stained for synaptophysin (cyan), synaptogyrin-3 (magenta) and tau (yellow). Representative images of maximum intensity z-projections of 5 serial sections are shown for a control (**a**) and a PSP case (**b**). The top row of each panel contains a 50 × 50 μm region of interest with a 10 μm scale bar, and the bottom rows contain a zoomed-in 10 × 10 μm region of interest with a 2 μm scale bar. The far-right column shows three-dimensional reconstructions of 5 serial sections. An arrow indicates a synapse containing tau in the PSP case. **c**–**f**, Quantification of segmented array tomography images reveals increases in the percentage of synaptogyrin-3 puncta containing tau (**c**, ANOVA after linear mixed effects model on Tukey transformed data ~ diagnosis + (1 | case/block) estimate = 0.279, 95% CI (0.004, 0.553), *t*(11.68) = 2.25, *p* = 0.044), synaptophysin puncta containing tau (**d**, estimate = 0.206, 95% CI (0.000037, 0.413), *F*(11.1) = 5.0167, *p* = 0.047) and increased tau burden (**e**, estimate = 0.427, 95% CI (0.251, 0.603), *F*(17.1) = 32.789, *p* < 0.0001). Tau burden positively correlates with the percentage of synaptogyrin-3 puncta containing tau in PSP but not control cases (**f**). Boxes show quartiles and medians calculated from each image stack, and whiskers show minima and maxima excluding outliers (n = 7 control, 7 PSP cases, 1–2 blocks per case, 2–3 stacks per block, group comparison statistics with linear mixed effects model). Correlations with Pearson’s correlation test. Data points refer to case means (females, circles; males, triangles).

To examine subsynaptic localization of tau, postmortem brain tissue from substantia nigra (SN) and frontal cortex of people who died with PSP and control donors was prepared for array tomography (details of donors are found in Extended Data Table 1). Ribbons of serial ultrathin sections were immunostained for phospho-tau Ser202/Thr205 (AT8) and synaptophysin (SYO; Fig. 1a). The proportion of synapses containing tau staining was analyzed with linear mixed-effect modeling of Tukey-transformed data (%_synapses_with_tau ~ diagnosis × brain_region + 1 | case_ID). We observe an accumulation of AT8-positive phospho-tau in synapses with a higher proportion in PSP brains than in controls (Fig. 1b). Post hoc analyses show this increase in colocalization of AT8 and SYO in PSP is significant in both the SN and cortex (Fig. 1b). Within PSP cases, we detected more AT8-positive synapses in the SN compared to the frontal cortex (Fig. 1b). As expected, stereological analyses of AT8 staining of formalin-fixed, paraffin-embedded (FFPE) sections revealed increased AT8 staining in PSP SN and frontal cortex when compared to control (Extended Data Fig. 1a,b). To validate our SYO antibody used in array tomography staining labels bona fide synapses, we used immunogold electron microscopy and indeed observed SYO immunogold labeling on synaptic vesicles in presynaptic terminals opposed to postsynaptic densities (Extended Data Fig. 1c,d). To test whether tau colocalizes with the presynaptic vesicle protein synaptogyrin-3 in human PSP synapses (as has been previously observed in mice and flies^8,24^), array tomography ribbons from the frontal cortex were also immunostained for total tau, synaptogyrin-3 and SYO (Extended Data Fig. 2a,b). We observed a fivefold increase in colocalization between total tau and synaptogyrin-3 in PSP (Extended Data Fig. 2c; median 0.16% of synapses in PSP and 0.03% in controls). Similarly, the percentage of SYO puncta containing total tau was significantly higher in PSP cases (Extended Data Fig. 2d; median 0.04% in PSP and 0% in controls). Spearman’s correlation reveals that the proportion of synaptogyrin-3 puncta containing tau is positively correlated with the tau burden in PSP cases but not in control cases (Extended Data Fig. 2f).

### Oligomeric tau accumulates within synaptic pairs in PSP

**Fig. 2:**
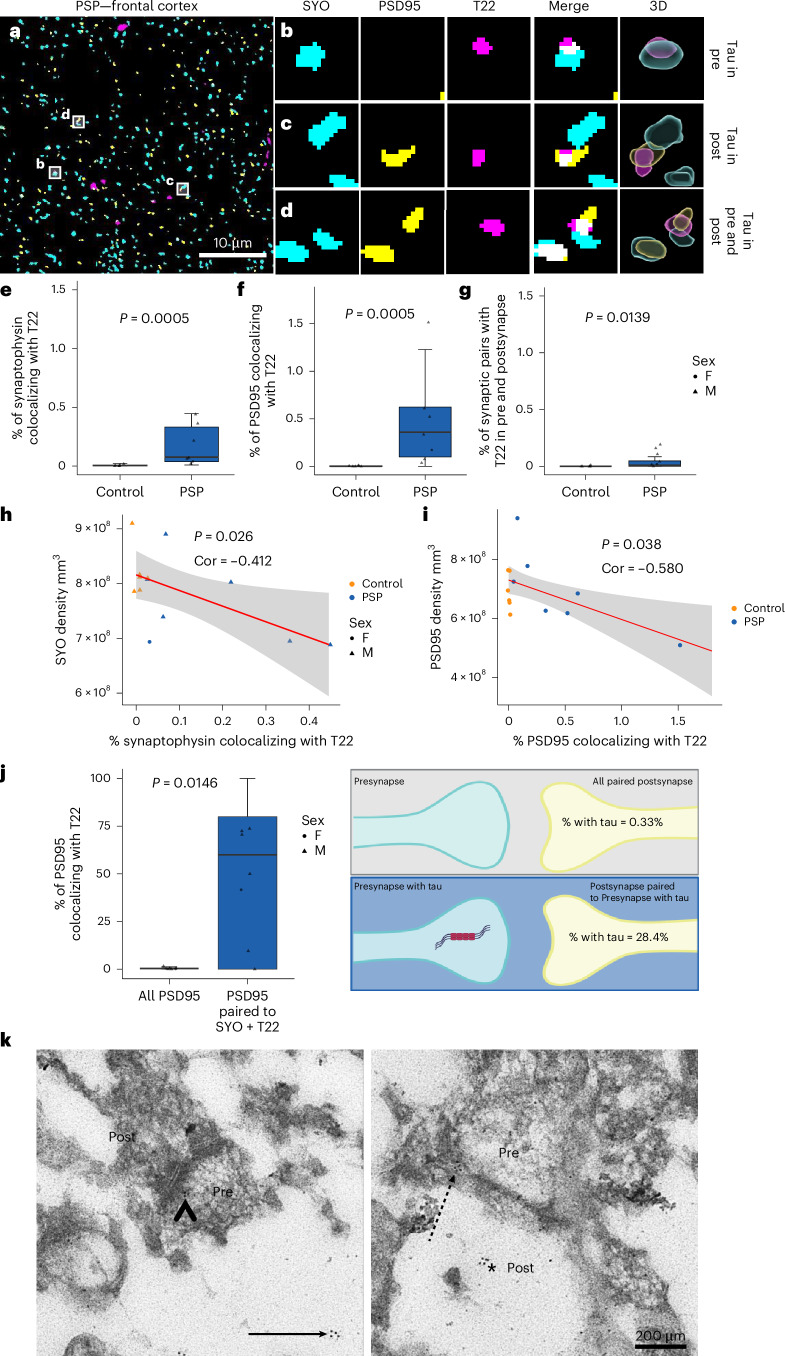
Indirect evidence for trans-synaptic spread and synaptotoxicity of oligomeric tau. **a**–**d**, A single 70 nm segmented section of PSP frontal cortex, immunostained for presynapses (SYO, cyan), postsynapses (PSD95, yellow) and oligomeric tau (T22, magenta). Scale bar = 10 µm. Oligomeric tau is seen in presynapses (**b**), postsynapses (**c**) and in both the presynapse and postsynapse of a synaptic pair (**d**). Boxes = 2 µm × 2 µm, 3D reconstructions made using IMARIS. **e**–**j**, Boxplots show the first quartile to third quartile (box) with the median marked, and whiskers show maximum and minimum values excluding outliers calculated from each image stack (two to three image stacks per case were taken as technical replicates). Data points show case means (*n* = 6 control, 7 PSP biological replicates). Data were Tukey transformed and analyzed with LMEMs (variable ~ diagnosis + 1 | case), and Tukey-corrected post hoc comparisons were performed. Quantification shows that in PSP frontal cortex there is an increase in the percentage of presynapses (**e**, estimate = 0.429, 95% CI (0.234, 0.624), *t*(10.7) = 4.85, *P* = 0.0005), postsynapses (**f**, estimate = 0.606, 95% CI (0.332, 0.88), *t*(10.6) = 4.89, *P* = 0.0005) and synaptic pairs colocalizing with oligomeric tau (**g**, estimate = 0.3, 95% CI (0.077, 0.522), *t*(8.9) = 3.052, *P* = 0.0139). The percentage of presynapses (**h**) and postsynapses (**i**) with oligomeric tau negatively correlates with the density of presynapses and postsynapses, respectively (Pearson correlations pre—*t*(27) = −2.348, 95% CI (−0.6763, −0.0533); post—*t*(27) = −2.857, 95% CI (−0.7210, −0.1400)). Postsynapses that are paired to a presynapse with oligomeric tau are around 86 times more likely to colocalize with oligomeric tau than all paired postsynapses, regardless of whether the adjacent presynapse also colocalizes with tau (**j**, estimate = 27.7, 95% CI (6.54, 48.8), *t*(11.8) = 2.856, *P* = 0.0146). Values shown in the schematic in **j** are means per group. Schematic created with biorender.com. **k**, Validation of oligomeric tau in synapses by immunogold electron microscopy. In postmortem PSP frontal cortex, anti-oligomeric tau (T22) immunogold staining is positive within the cytoplasm (arrow), presynaptic vesicles (arrowhead), oligomer-like structures in postsynapses (asterisk) and at the synaptic cleft (dotted arrow). Scale bar = 200 nm.

**Extended Data Fig. 3:**
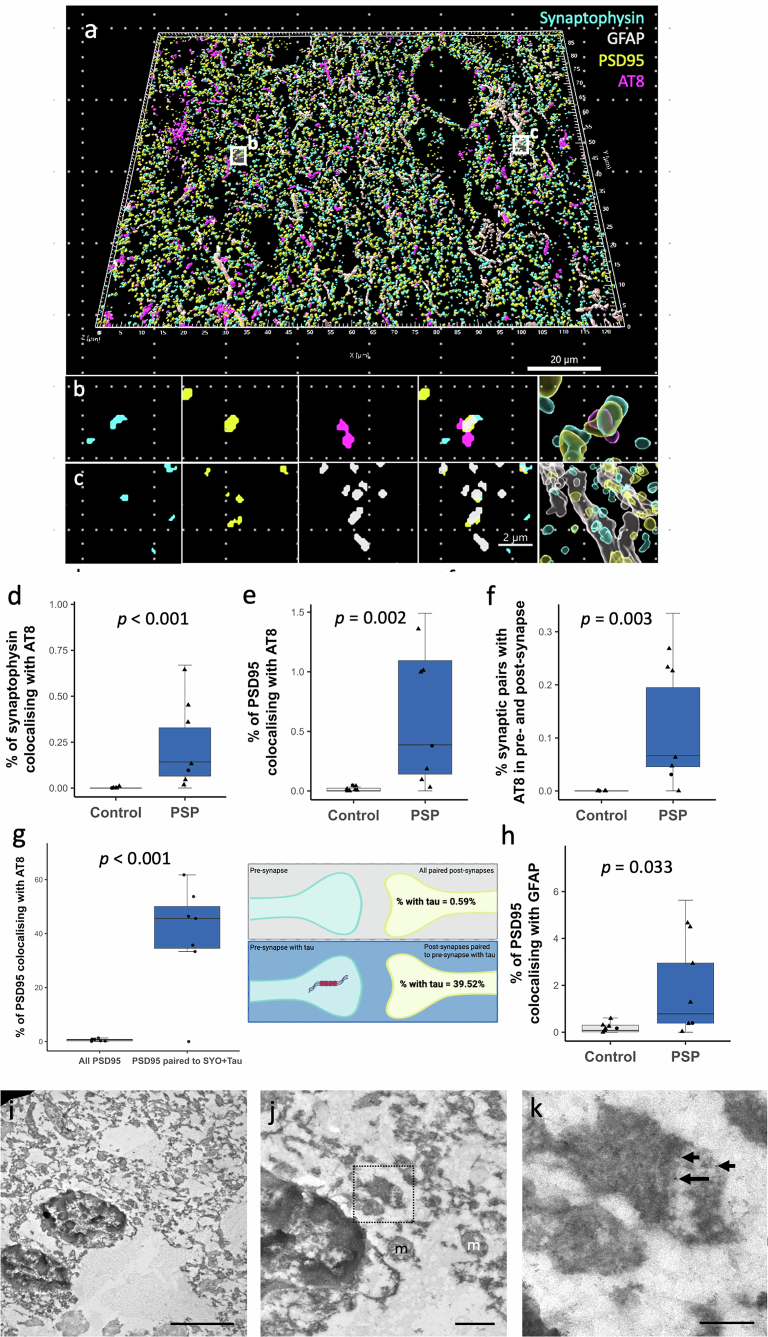
Evidence for the trans-synaptic spread of phospho-tau 202/205, augmented post-synaptic engulfment by astrocytes in PSP frontal cortex and electron microscopy validation of microglial ingestion by synapses. **a**–**c**, A 3D reconstruction of PSP frontal cortex, immunostained for pre-synapses (synaptophysin, cyan), post-synapses (PSD95, yellow), tau (AT8, magenta) and astrocytes (GFAP, gray; **a**). Tau (AT8) is seen to accumulate in synaptic pairs (**b**) and pre-synapses and post-synapses are seen within GFAP-positive astrocyte processes (**c**). 3D reconstructions in **a** and far-right panels of **b** and **c** were made using IMARIS. **d**–**h**, Quantification shows that in PSP frontal cortex there is an increase in the percentage of pre-synapse (**d**, ANOVA after linear mixed effects model on Tukey transformed data ~ cohort + age + 1 | case, estimate = 0.519, 95% CI (0.305, 0.734), *F*(11) = 17.559, *p* < 0.0001), post-synapses (**e**, estimate = 0.565, 95% CI (0.268, 0.861), *F*(11) = 28.441, *p* = 0.002) and synaptic pairs (**f**, estimate = 0.32, 95% CI (0.137, 0.502), *F*(11) = 14.7803, *p* = 0.003) colocalizing with tau (AT8). Post-synapses that are paired to a pre-synapses with tau (AT8) are 67.1 times more likely to colocalize with tau (AT8) than all paired post-synapses, regardless of whether the adjacent pre-synapse also colocalizes with tau (**g**, estimate = 0.787, 95% CI (0.076, 1.5), *F*(21.649) = 39.885, *p* < 0.0001). There is also an increased colocalization between post-synapses (PSD95) and astrocytes (GFAP; **h**, estimate = 29.9, 95% CI (14.2, 45.6), *F*(11) = 5.939, *p* = 0.033). Boxes show quartiles and medians calculated from each image stack; whiskers show minima and maxima excluding outliers. Quantifications are from n = 7 PSP, 7 control cases with 2 image stacks per case as technical replicates. Data points show case means (biological replicates—females, circles; males, triangles). Schematic created with biorender.com. **i**–**k**, Immunoelectron microscopy was used to confirm synaptic ingestion by microglia. Microglia were identified by ultrastructure, including dark nuclei (nuc). Higher power images of the microglial cytoplasm (**j**,**k**) show mitochondria (m) and cytoplasmic structures. Higher magnification images of synaptophysin labeled with 10 nm gold particles arrows in (**k**, inset shown in **j**) show synaptic vesicle-containing structures in the microglial cytoplasm. Scale bars = 3 μm in **i**, 500 nm in **j** and 200 nm in **k**.

We further used array tomography to investigate the synaptic distribution of oligomeric tau (T22 antibody, validated in ref. ^30^) in the frontal cortex. The frontal cortex was chosen for this experiment as it is a brain region that is generally affected later by neuronal tau pathology than subcortical regions^2^. Our reasoning was that if trans-synaptic tau spread is a mechanism driving tau pathology progression, then a greater proportion of the tau present in the frontal cortex may have spread there, compared to an early affected region where disease initiation occurs. Array tomography ribbons were stained for oligomeric tau (T22), the presynaptic vesicle protein SYO and postsynaptic density protein 95 (PSD95; Fig. 2a). Colocalization analyses were performed, and we observed that, compared to control, there is an increased proportion of presynapses (Fig. 2b,e) and postsynapses (Fig. 2c,f) colocalizing with oligomeric tau in PSP. In total, 6.42% of the T22 staining in the neuropil (excluding somatic aggregates) colocalized with SYO and 18.6% colocalized with PSD95, showing that together over 25% of oligomeric tau in the neuropil is located within synapses. Interestingly, there was a negative correlation between the percentage of oligomeric tau-containing presynapses (Fig. 2h) and postsynapses (Fig. 2i) and their respective synaptic density, indicating that oligomeric tau is synaptotoxic. In PSP brain, we also observed an increase in synaptic pairs where tau oligomers were observed within both the presynapse and the opposing paired postsynapse (Fig. 2d,g). Synaptic pairs were defined as PSD95 puncta with a SYO puncta within 0.5 μm (distance between centroids) as described previously^10^. To investigate whether tau within both sides of a synaptic pair could be through random chance, we examined whether postsynapses were more likely to contain tau if they were opposed to a presynapse that also contains tau. In PSP brain, we found that postsynapses paired to a presynapse containing oligomeric tau were 86 times more likely to colocalize with tau than all paired postsynapses regardless of the tau status of the paired presynapse (Fig. 2j). We confirmed the presence of tau oligomers within presynapses and postsynapses in PSP frontal cortex using immunogold electron microscopy (Fig. 2k). We found similar evidence of synaptic phospho-tau accumulation when using array tomography to examine the presence of AT8 in synaptic pairs (Extended Data Fig. 3a–g).

### Astrocytes may contribute to synaptic loss in PSP

**Fig. 3:**
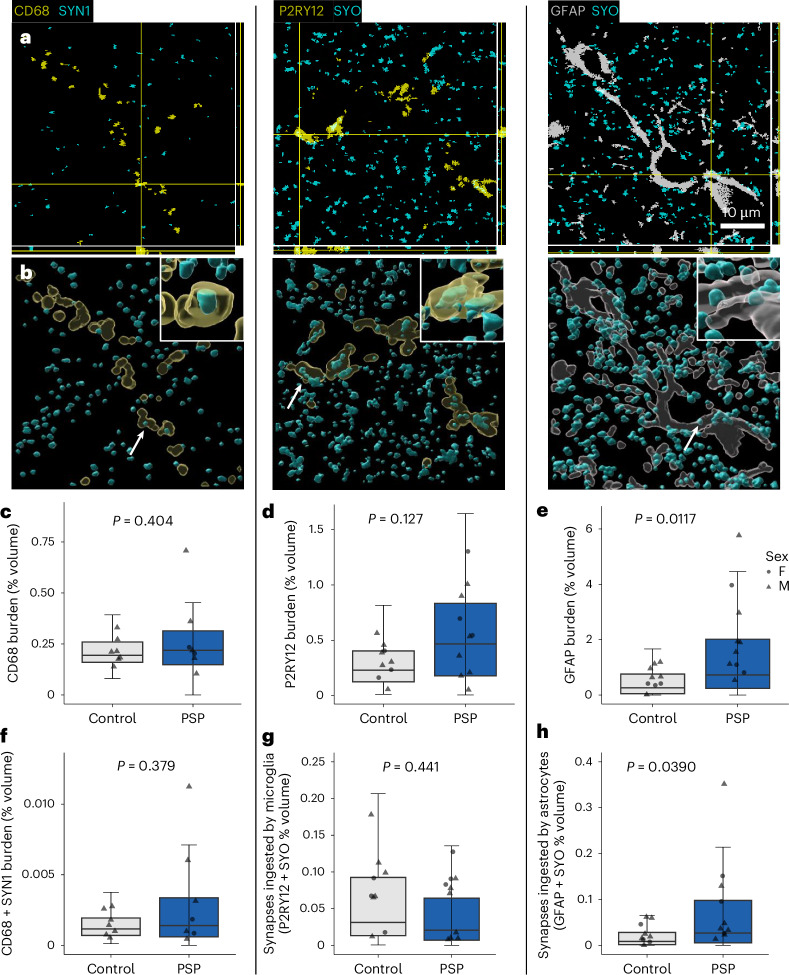
Increased synaptic engulfment by astrocytes but not microglia in PSP frontal cortex. **a**, Confocal microscopy of PSP frontal cortex stained with GFAP to label astrocytes (gray); CD68 or P2RY12 to label microglia (yellow); SYN1 or SYO (cyan) to label synapses. Orthogonal views are shown to demonstrate colocalization between synaptic protein and glia. Boxes = 50 μm × 50 μm. Scale bar = 10 μm. **b**, Three-dimensional reconstructions of image stacks. Insets show close-ups of the colocalizations highlighted by the arrow, which have been rotated along the *z* axis, to further demonstrate that synaptic protein is within glia. Three-dimensional reconstructions made in IMARIS. Large boxes = 50 μm × 50 μm, insets = 5 μm × 5 μm. **c**–**h**, Quantitative analysis using linear mixed effects modeling on Tukey transformed data (variable ~ diagnosis + 1 | case) and post hoc Tukey-corrected pairwise comparisons reveals that there is no difference in the burden (percentage of volume of image stack occupied by staining) of CD68 (**c**, *n* = 7 PSP, 7 control cases with ten image stacks per case as technical replicates, estimate = 0.069, 95% CI (−0.1, 0.243), *t*(12) = 0.865, *P* = 0.404) or P2RY12 (**d**, *n* = 10 PSP, 9 control cases with ten image stacks per case as technical replicates, estimate = 0.131, 95% CI (−0.0411, 0.303), *t*(17) = 1.607, *P* = 0.127) in PSP frontal cortex when compared to control. Furthermore, there is no difference in the amount of colocalization between CD68 and SYN1 (**f**, *n* = 7 PSP, 7 control cases with ten image stacks per case as technical replicates, estimate = 0.0248, 95% CI (−0.0344, 0.084), *t*(12) = 0.913, *P* = 0.379) and P2RY12 and SYO (**g**, *n* = 10 PSP, 9 control cases with ten image stacks per case as technical replicates, estimate = 0.036, 95% CI (−0.133, 0.06), *t*(16.9) = 0.789, *P* = 0.441). There is an increase in GFAP burden (**e**, *n* = 10 PSP, 9 control cases with ten image stacks per case as technical replicates, estimate = 0.186, 95% CI (0.047, 0.325), *t*(16.8) = 2.826, *P* = 0.0117) and colocalization between GFAP and SYO (**h**, estimate = 0.127, 95% CI (0.00719, 0.247), *t*(16.9) = 2.238, *P* = 0.0390) in PSP frontal cortex when compared to control. Boxes show quartiles and medians calculated from each image stack. Whiskers show minima and maxima of stack values without outliers. Data points show case means.

To investigate the presence of astrogliosis, microgliosis and synaptic engulfment by astrocytes and microglia in PSP, we immunostained 4-µm-thick FFPE sections from PSP and control frontal cortex with antibodies against astrocytes (glial fibrillary acidic protein, GFAP), microglia (CD68 or P2RY12), presynapses (synaptophysin (SYO) or synapsin-1 (SYN1)) and phosphorylated tau (AT8; Fig. 3a,b). The burden (percentage of volume of image stack occupied by staining) of individual channels and the colocalization between channels were calculated. There is an increase in GFAP staining (Fig. 3e) and an increase in GFAP and SYO colocalization (Fig. 3h). There was no difference between PSP and control CD68 burden (Fig. 3c), P2RY12 burden (Fig. 3d), colocalization between CD68 and SYN1 (Fig. 3f) or colocalization between P2RY12 and SYO (Fig. 3g).

**Fig. 4:**
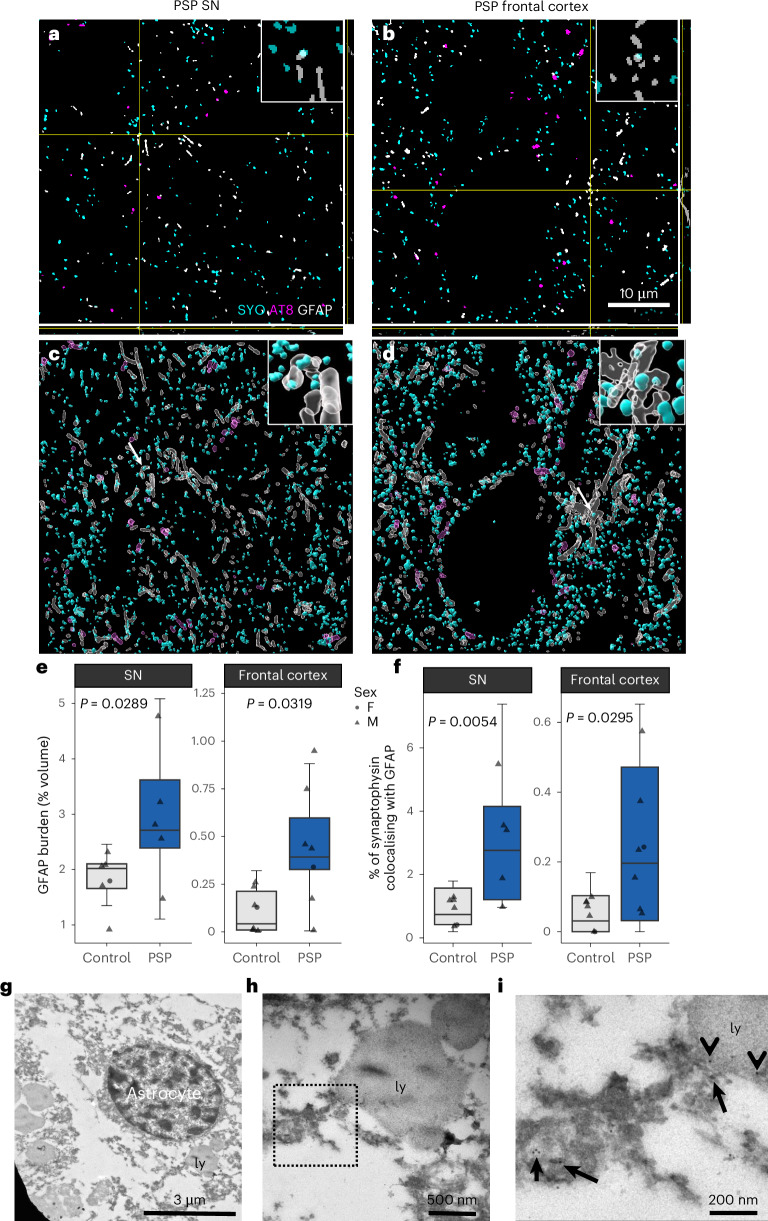
Increased synaptic engulfment by astrocytes in PSP SN and frontal cortex demonstrated using array tomography and immunogold electron microscopy. **a**–**d**, A single 70 nm segmented array tomography section with orthogonal views of the image stack (**a**,**b**), and 3D reconstructions (**c**,**d**) of PSP SN (**a**,**c**) and frontal cortex (**b**,**d**) immunostained for presynapses (SYO, cyan), tau (AT8, magenta) and astrocytes (GFAP, gray). Arrows and insets show colocalization between synapses (SYO) and astrocytes (GFAP). Large boxes = 50 μm × 50 μm, scale bar = 10 μm. Small boxes = 5 μm × 5 μm. **e**,**f**, Array tomography image stacks were taken from PSP (*n* = 5 donors SN, 7 frontal cortex) and control (*n* = 6 SN, 7 frontal cortex). Two to three image stacks per region per case were taken as technical replicates. Boxplots show the first quartile to third quartile (box) with the median marked, and whiskers show maximum and minimum values calculated from each image stack, excluding outliers. Data points show case means (biological replicates). Data were analyzed with LMEMs (~diagnosis × brain region + 1 | case) with post hoc pairwise Tukey-corrected comparisons. Quantification reveals that in PSP SN and frontal cortex, there is astrogliosis (**e**, SN estimate = 0.334, 95% CI (0.039, 0.629), *t*(16.6) = 2.393, *P* = 0.0289; frontal cortex estimate = 0, 95% CI (0.0316, 0.601), *t*(14.1) = 2.382, *P* = 0.0319, square root transformed data LMEM ~ diagnosis × brain_region + 1 | case). Further in PSP, there is increased colocalization between presynapses (SYO) and astrocytes (GFAP; **f**, model run on Tukey transformed data, SN estimate = 0.360, 95% CI (0.116, 0.60), *t*(26.8) = 3.029, *P* = 0.0054, frontal cortex estimate = 0.243, 95% CI (0.026, 0.459), *t*(23.2) = 2.320, *P* = 0.0295). **g**–**i**, Immunogold electron microscopy of human postmortem PSP frontal cortex with anti-SYO antibody labeled with 10 nm gold particles. Astrocytes were identified by ultrastructure (**g**) and often included lysosomes (ly). A higher magnification image of the astrocytic cytoplasm in the human brain (**h**) shows phagocytosed debris, including membranous structures abutting lysosomes. Higher magnification image (**i**) shows synaptic vesicle-containing structures (arrows) labeled for SYO with 10 nm gold in the astrocytic cytoplasm and gold particles in the lysosome (arrowheads). Scale bars = 3 µm (**g**), 500 nm (**h**) and 200 nm (**i**).

The resolution of confocal microscopy of these images of 4-µm-thick paraffin tissue sections is limited by the diffraction limit of light. At this resolution, it is not possible to distinguish individual synapses. Furthermore, research using correlative light and electron microscopy suggests that the majority of synapse encapsulation by microglia observed using confocal microscopy of thick sections is actually synapse–glia apposition and not internalization of synapses by glia^31^. Therefore, to confirm the presence of synaptic engulfment by astrocytes in PSP SN and frontal cortex at higher resolution, we immunostained array tomography ribbons for GFAP, SYO and AT8 (Fig. 4a–d). We observe an increase in GFAP burden in both the SN and frontal cortex consistent with the FFPE data (Fig. 4e). There is also an increase in the percentage of presynapses colocalizing with GFAP-positive astrocytes in the SN and frontal cortex (Fig. 4f). In some PSP cases, SYO colocalized with both GFAP and AT8, suggesting that astrocytes might be engulfing tau-containing synapses. In the frontal cortex, this analysis was repeated with PSD95 with comparable results (Extended Data Fig. 3h), suggesting that in the frontal cortex of PSP brain, astrocytes are engulfing an increased number of both presynapses and excitatory postsynapses. Furthermore, using immunogold electron microscopy with an anti-SYO antibody, we confirmed the presence of synaptic material within astrocytes and astrocytic lysosomes in PSP brain (Fig. 4g–i).

### Synaptic, inflammatory and metabolic protein changes in PSP

**Fig. 5:**
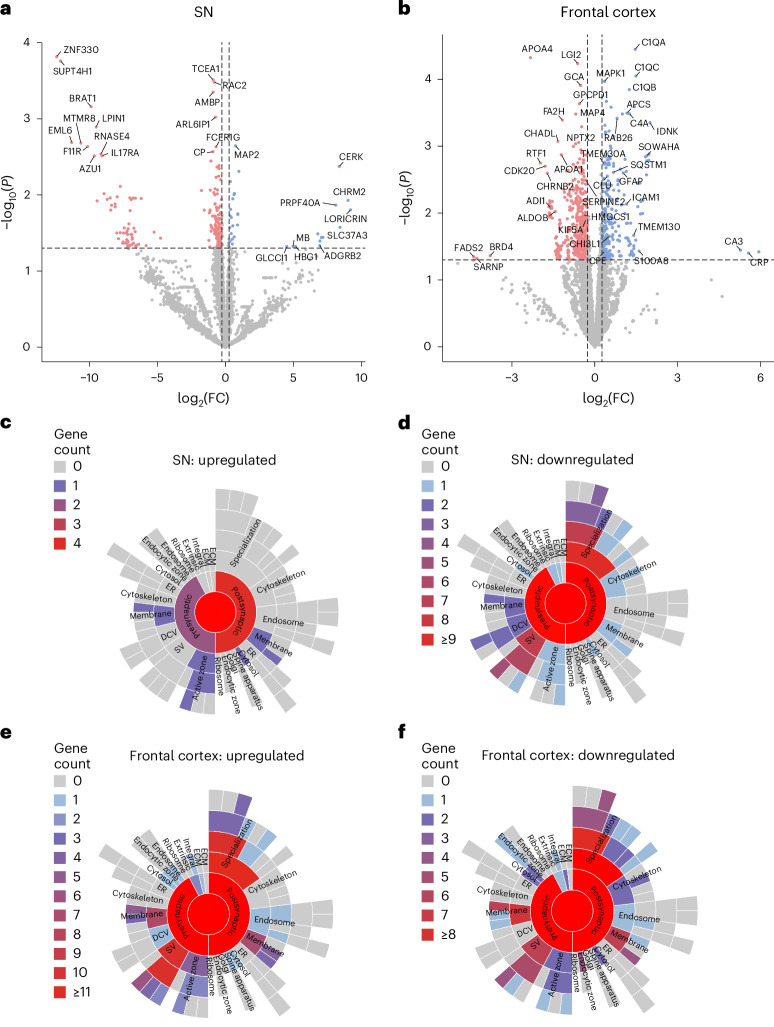
Proteomics analysis identifies dysregulated synaptic proteins. **a**,**b**, Volcano plots highlighting the results of proteomic analysis comparing synaptic enriched fractions from PSP and control SN (**a**) and frontal cortex (**b**). DEPs were defined as *P* < 0.05 and a 20% change in protein level. Downregulated proteins are shown in red. Upregulated proteins are shown in blue. Proteins that were not differentially expressed are highlighted in gray. SynGO analysis was conducted on the synaptic enriched fraction preparations from the SN (**b**,**c**) and frontal cortex (**d**,**e**). **c**,**d**, In the SN, there are very few upregulated proteins (**c**) and a substantial loss of presynaptic and postsynaptic proteins (**d**). **e**,**f**, In the frontal cortex, there are similar numbers of upregulated (**e**) and downregulated (**f**) proteins.

**Extended Data Fig. 4:**
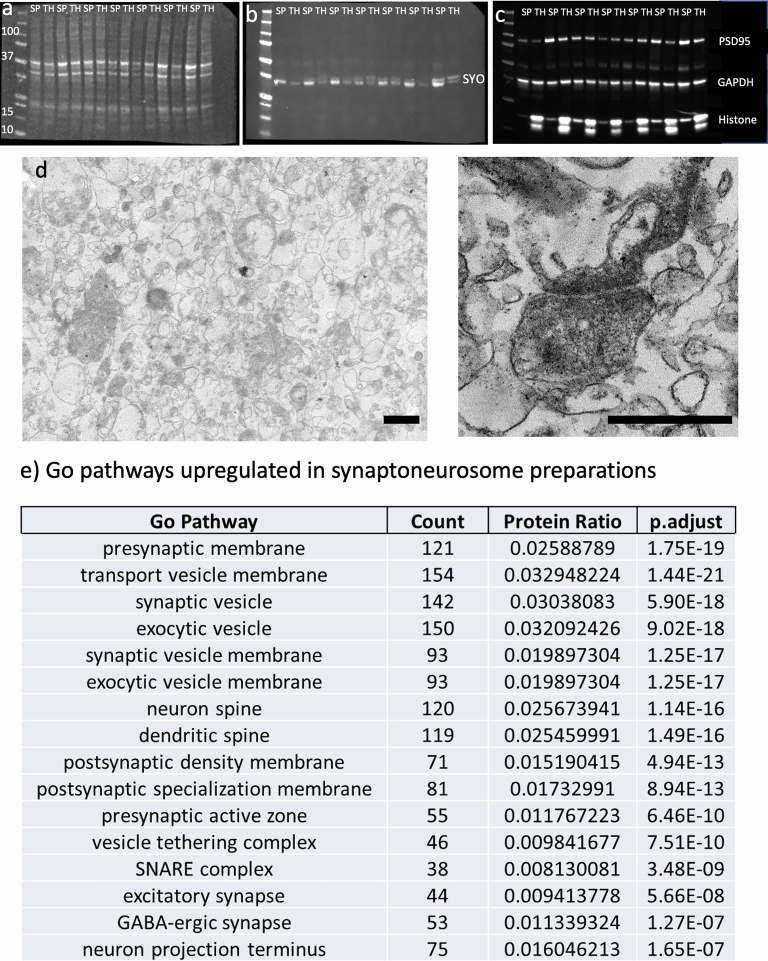
Validation of enrichment of synaptic proteins in synaptic enriched fraction preparations. **a**–**c**, Western blot analysis reveals enriched synaptic proteins (synaptophysin and PSD95) and decreased nuclear protein (histone H3) in synaptic enriched fraction preparations. Membrane was probed with REVERT total protein stain (**a**), synaptophysin (SYO; **b**) and PSD95, histones H3 and GAPDH (**c**). SP, synaptic preparation; TH, total homogenate. **d**, Electron microscopy of synaptic preparations shows exclusion of cell bodies and enrichment of synapses. Scale bar left image = 1 μm; scale bar right image = 0.5 μm. **e**, Gene Ontology enrichment analysis using the R package ‘clusterProfiler’, setting = ‘cellular compartment’, was performed on DEPs between total homogenate and synaptic preparations and revealed an enrichment of synaptic proteins (**e**).

**Extended Data Fig. 5:**
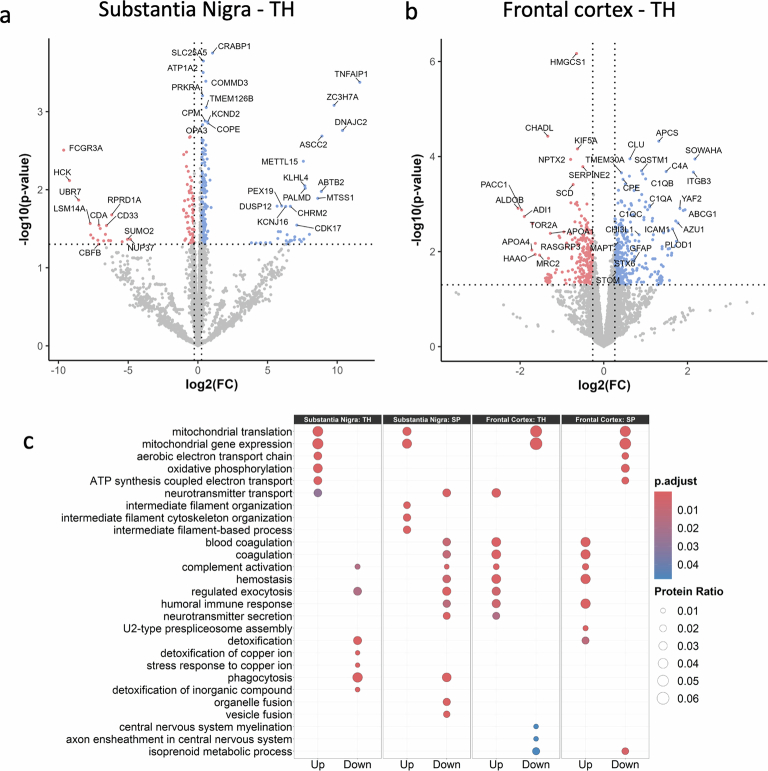
Differentially expressed proteins in total homogenates of PSP substantia nigra and frontal cortex. **a**,**b**, Volcano plots highlighting the results of proteomic analysis comparing PSP and control substantia nigra total homogenate (**a**) and frontal cortex total homogenate (**b**). Differentially expressed proteins (DEPs) were defined as *p* value < 0.05 and a 20% change in protein level. Downregulated proteins are shown in red. Upregulated proteins are shown in blue. Proteins that were not differentially expressed are highlighted in gray. TH, total homogenate. **c**, Gene Ontology enrichment analysis using the R package ‘clusterProfiler’ (setting: ‘biological processes’) was performed on DEPs, highlighting that synaptic, metabolic and inflammatory pathways are altered in PSP. *P* value adjusted using Benjamini–Hochberg correction for multiple comparisons.

To investigate molecular changes in remaining synapses in PSP and look for candidate proteins that might be involved in trans-synaptic tau spread, mass-spectroscopy proteomics was performed on soluble brain homogenates and synaptic enriched fractions from the SN and frontal cortex of PSP and control cases. To confirm that our synaptic preparations were effective, we used western blots to confirm enrichment of synaptic proteins SYO and PSD95 and exclusion of nuclear histone protein (Extended Data Fig. 4a–c). Furthermore, we confirmed predominantly synaptic structures using electron microscopy (Extended Data Fig. 4d), and finally, Gene Ontology (GO) analysis of cellular compartments present confirmed that our synaptoneurosome (SN) preparations were enriched for multiple synaptic proteins when compared to the total brain homogenate (TH) preparations (Extended Data Fig. 4e). The proteomes were then compared between PSP and control frontal cortex and SN. Volcano plots illustrate the extent of protein changes detected between PSP and control (Fig. 5 and Extended Data Fig. 5). Differentially expressed proteins (DEPs) of interest include the following: the complement proteins C4A, C1QA and C1QC; the neuroinflammatory proteins GFAP, YKL40/CHI3L1 and ICAM1; the synaptic protein syntaxin-6 (STX6), stomatin and clusterin. Interestingly, tau protein levels were not different between PSP and control in synaptic fractions or total homogenates, indicating that soluble total tau levels were unchanged in disease.

To interrogate synaptic changes further, the synaptic GO tool, SynGO, was used to examine changes in levels of known synaptic proteins stratified by their typical synaptic localization (Fig. 5). In PSP SN, there was evidence for a pan-synaptic loss of proteins compared to controls. Decreases were observed in proteins associated with the presynapse (including SNAPIN, SYT5 and NCAM1), synaptic cleft (NPTX1) and postsynapse (including NLGN1, HOMER3 and NCAM1; Fig. 5c,d). Using SynGO to identify the biological processes associated with these synaptic proteins identifies dysregulation of the following synaptic processes in PSP SN: the synaptic vesicle, the regulation of postsynaptic membrane potential, the regulation of postsynaptic neurotransmitter receptor activity, trans-synaptic signaling and synapse organization. There were only a total of five synaptic proteins upregulated in PSP SN; this includes one presynaptic protein (CHRM2) associated with trans-synaptic signaling, four postsynaptic proteins (APC2, MAP2, ALDOC and CHRM2) and ELAVL4, an RNA-binding protein involved in translation at the synapse. While in the SN, the majority of synaptic DEPs were downregulated, in the frontal cortex, there was evidence for both loss of some synaptic proteins and increases in others (Fig. 5e,f). The downregulated proteins in PSP frontal cortex were associated with the synaptic membrane (CADM4), the presynapse (including BIN1 and SNAP25), the synaptic cleft (NPTX2 and NXPH1) and the postsynapse (including ADAM10, BIN1, GABARAP and NPTX2). The upregulated proteins in PSP frontal cortex were associated with the synaptic membrane (ANXA1 and ANO6), the presynapse (including GABRA3, SYN1 and SYT5), the synaptic cleft (LAMC1) and the postsynapse (including GABRA3, CRTC1, MAPK1 and MAP2K1). These upregulated synaptic proteins are involved in the regulation of presynaptic cytosolic calcium levels, the regulation of synaptic membrane potential, synaptic vesicle exocytosis and endocytosis, the regulation of postsynaptic membrane neurotransmitter receptor levels, trans-synaptic signaling, synapse organization, synapse assembly and synapse metabolism.

For a deeper understanding of the biological and synaptic pathways affected in PSP, DEPs were analyzed using GO enrichment (setting—biological process)^32^ (Extended Data Fig. 5). When analyzing TH, alterations were evident in several biological pathways in PSP, including changes in metabolic pathways such as mitochondrial translation and gene expression, oxidative phosphorylation, aerobic transport chain and ATP synthesis. Alterations in immune and inflammatory pathways, such as complement activation, humoral immune response, detoxification and stress response, were also observed. Dysregulation of synaptic and signaling pathways, such as neurotransmitter transport, neurotransmitter signaling and vesicle fusion, was evident. Interestingly, while the same pathways were often altered in both the frontal cortex and SN, the direction of change was often reversed (Extended Data Fig. 5). This observation suggests a potential dichotomous response in end-stage and actively compensatory stages of the disease.

### Clusterin and oligomeric tau colocalize within PSP synapses

**Fig. 6:**
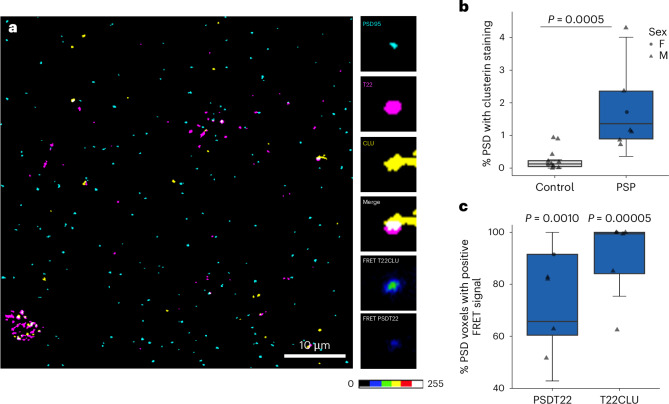
Oligomeric tau is close enough to clusterin to be a binding partner within postsynapses in PSP brain. **a**, A single 70 nm segmented array tomography section of PSP frontal cortex immunostained for oligomeric tau (T22, magenta), clusterin (CLU, yellow) and postsynapses (PSD95, cyan). Scale bar = 10 μm. Insets show colocalization and positive FRET signal between clusterin and oligomeric tau and oligomeric tau and PSD95 within a postsynapse. Scale bar for FRET images indicates intensity (0–255 a.u.). **b**, As predicted from the proteomics results, colocalization of clusterin and PSD95 was higher in PSP than in control (PSP, *n* = 7 control and *n* = 10 cases, two to four image stacks per case as technical replicates, LMEM on Tukey transformed data ~ diagnosis + (1 | case), Tukey-corrected post hoc pairwise comparison estimate = 0.646, 95% CI (0.323, 0.956), *t*(12.2) = 4.743, *P* = 0.0005). **c**, In PSP frontal cortex, PSD95 and T22 generate a FRET signal significantly different from 0 (one-sample *t*-test on means per case with *μ* = 0, *t* = 10.12, d*f* = 4, Bonferroni-corrected *P* = 0.0010). Similarly, within postsynapses positive for both T22 and clusterin, these proteins generate a FRET signal (*t*(5) = 14.644, Bonferroni-corrected *P* = 0.00005), indicating these proteins are likely interacting within synapses. Boxes show the first quartile, median and third quartile of the stack data. Whiskers show minima and maxima without outliers. Data points show case means (females, circles; males, triangles).

Our proteomics analysis revealed that clusterin was increased by 1.37-fold in frontal cortex synapses in PSP, which is of interest as clusterin gene variants are associated with AD risk, and our previous data implicate clusterin in amyloid β-mediated synapse loss in AD^33^. To investigate whether clusterin may also be involved in mediating synaptic tau pathology, we immunostained array tomography ribbons from PSP and control frontal cortex with antibodies against postsynapses (PSD95), oligomeric tau (T22) and clusterin (Fig. 6a). Colocalization analysis confirmed that there is increased colocalization between postsynapses (PSD95) and clusterin in PSP (Fig. 6b). Förster resonance energy transfer (FRET) was used to identify proteins within 10 nm of each other. Within PSP brain, when looking at the postsynapses that colocalize with oligomeric tau, there is a positive FRET signal between these proteins (Fig. 6c). Furthermore, when looking at postsynapses that colocalize with both oligomeric tau and clusterin, there is a positive FRET signal between oligomeric tau and clusterin (Fig. 6c).

### Synapses take up oligomeric tau in live human brain slices

**Fig. 7:**
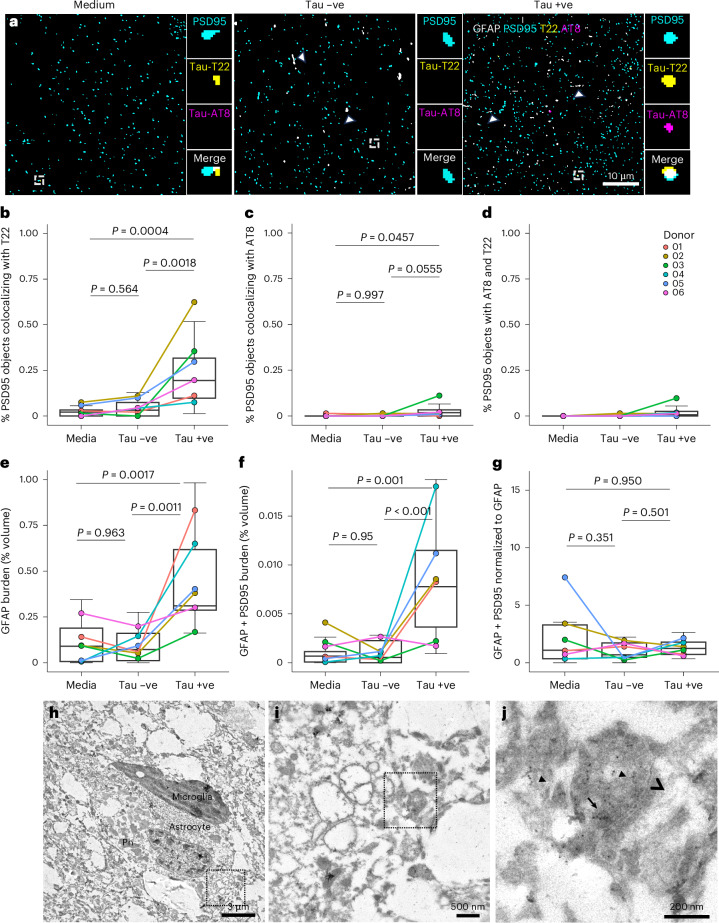
PSP-derived tau induces astrogliosis, synaptic engulfment and colocalizes with postsynapses in living human brain slices. **a**, Human brain slices were cultured for 72 h in either medium only, Tau −ve soluble PSP brain extract or Tau +ve extract. Slices were processed for array tomography and immunostained for postsynapses (PSD95, cyan), oligomeric tau (T22, yellow), phospho-tau Ser202, Thr205 (AT8, magenta) and astrocytes (GFAP, gray). Representative single 70 nm segmented sections are shown. Scale bar = 10 μm. Large boxes = 50 μm × 50 μm. Small boxes = 2 μm × 2 μm. Arrowheads show GFAP-positive astrocyte processes. Array tomography image stacks were taken from six human brain tissue donors (biological replicates), each treated with three conditions including two stacks (technical replicates) per condition, and Tukey-transformed data were analyzed using LMEMs (~treatment + 1|donor) followed by Tukey-corrected pairwise post hoc tests. **b**–**g**, Quantification of colocalization shows that incubating in Tau +ve PSP extract causes an increase in the percentage of PSD95 puncta with oligomeric tau (**b**), compared to either slices cultured in medium (estimate = 0.5146, 95% CI (0.28, 075), *t*(9.56) = 6.07, *P* = 0.0004) or compared to Tau −ve PSP brain extract (estimate = 0.423, 95% CI (0.19, 0.66), *t*(9.56) = 4.91, *P* = 0.0018). There is a trend toward an increase with AT8 accumulation when incubated with the Tau +ve PSP extract (**c**), compared to slices cultured in medium alone (estimate = 0.138, 95% CI (0.003, 0.273), *t*(13.4) = 2.68, *P* = 0.0457) or compared to Tau −ve PSP extract (estimate = 0.133, 95% CI (0.003, 0.270), *t*(13.4) = 2.57, *P* = 0.0555). There was no difference in the percentage of PSD95 puncta containing both AT8 and T22 (**d**) when cultured with the Tau +ve PSP extract. Slices cultured with the Tau +ve extract show an increase in the percentage volume of the 3D image stack occupied by GFAP (**e**) compared to medium alone (estimate = 0.35, 95% CI (0.14, 0.56), *t*(13) = 4.48, *P* = 0.0017) or compared to tau-immunodepleted PSP extract treatment (estimate = 0.37, 95% CI (0.16, 0.58), *t*(13) = 4.69, *P* = 0.0011) and an increase in the colocalization of GFAP with PSD95 (**f**) compared to medium alone (estimate = 0.114, 95% CI (0.05, 0.18), *t*(12) = 4.75, *P* = 0.001) and compared to Tau −ve PSP extract (estimate = 0.122, 95% CI (0.06, 0.19), *t*(12) = 5.00, *P* < 0.001). However, when the colocalization between PSD95 and GFAP is normalized to the GFAP burden, there is no difference between groups (**g**). Boxplots show quartiles and medians calculated from each image stack; whiskers show minima and maxima without outliers. Data points refer to donor case means. **h**–**j**, Immunogold electron microscopy of HBSC exposed to Tau +ve PSP extract, immunolabeled with anti-SYO antibody labeled with 10 nm gold particles. Astrocytes were identified by ultrastructure (**h**) and often included phagocytosed material (Ph). Higher magnification images (**i**,**j**) show SYO labeled with gold particles (arrows) opposed to a postsynaptic density (arrowhead) within the astrocyte cytoplasm. Scale bars = 3 µm (**h**), 500 nm (**i**) and 200 nm (**j**).

**Extended Data Fig. 6:**
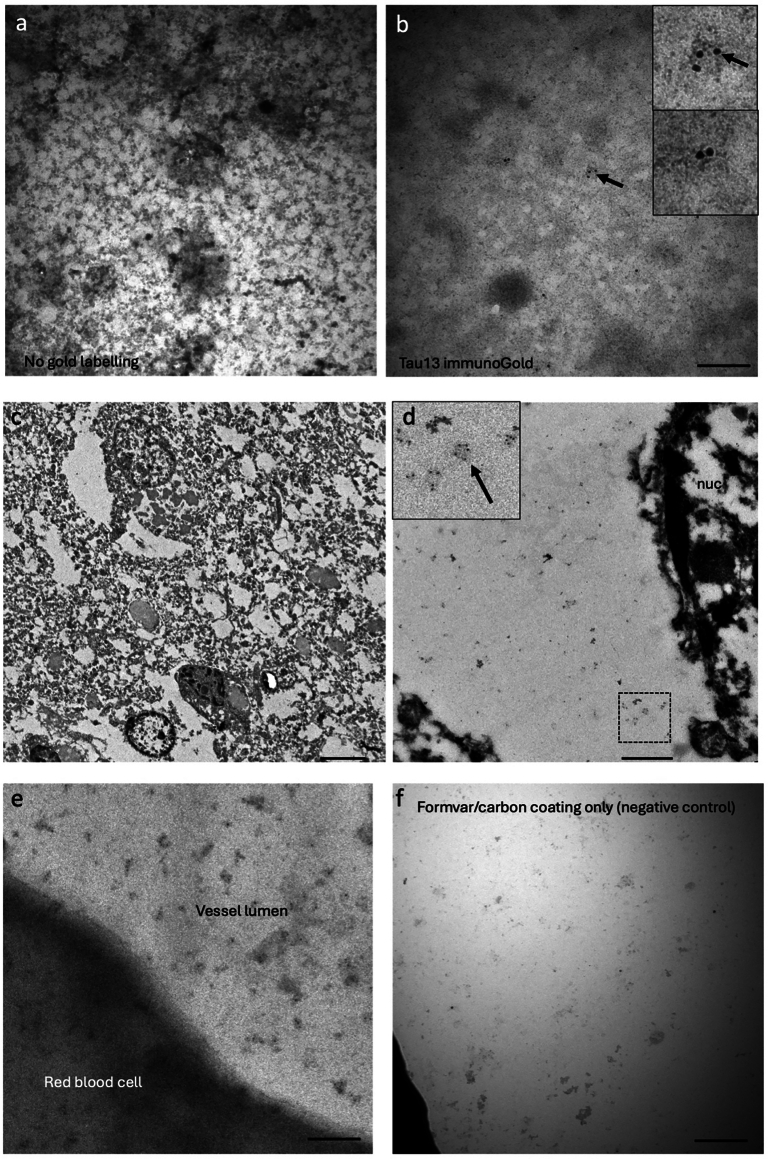
Characterization of oligomeric tau by electron microscopy. **a**, Recombinant tau oligomers negatively stained with uranyl acetate and lead citrate form globular structures. **b**, Tau +ve PSP brain extract used for slice culture experiments was incubated on Formvar/carbon-coated nickel electron microscope grids, and T22 was used for immunogold labeling of oligomeric tau. This shows similar globular structures to recombinant tau oligomers and no fibrils, as expected for this soluble brain extract. Arrows indicate 10 nm gold particles labeling oligomers. Scale bar for **a** and **b** = 200 nm, insets = 150 × 150 nm. **c**, Overview of ultrastructure of postmortem PSP brain stained with T22 immunogold. Scale bar = 5 μm. **d**, Higher magnification image of PSP post-mortem brain stained with T22 immunogold showing that in addition to synaptic oligomeric tau shown in the main figures, we also observe globular oligomeric structures labeled with T22. Arrow indicates a globular oligomer labeled with 10 nm gold particles. Scale bar = 500 nm, inset = 500 × 500 nm. **e**, Imaging of a blood vessel lumen and red blood cell. **f**, Imaging of blank areas of the grid with only Formvar/nickel coating acts as a negative control for immunogold staining. Scale bars for **e** and **f** = 200 nm.

To study the effects of PSP-derived tau on the living human brain, we used our recently developed slice culture model system using living adult human neocortex donated from surgical resections^34^ (donor information in Extended Data Table 1), which was challenged with postmortem soluble PSP brain extract. Immunogold electron microscopy of soluble PSP brain homogenate stained with an antibody that recognises total tau revealed the absence of large fibrillar tau structures, as expected in the soluble fraction. Tau-positive gold labeling was seen on small globular structures of similar size and appearance to tau oligomers (Extended Data Fig. 6)^35^. Human brain slices were cultured for 72 h in three different conditions. Group 1 was cultured in 100% medium (control), group 2 in 75% medium and 25% tau-immunodepleted PSP brain soluble protein extract (Tau −ve) and group 3 in 75% medium and 25% mock immunodepleted (thus, tau-containing (Tau +ve)) soluble PSP brain extract. Adjacent slices derived from the same patient’s brain tissue were treated with each of the three conditions, providing an internal control for each patient. After treatment, slices were collected for array tomography. Array tomography ribbons were immunostained for GFAP-positive astrocytes, postsynapses (PSD95), phospho-tau Thr202, Ser205 (AT8) and oligomeric tau (T22; Fig. 7a). Slices cultured with the Tau +ve soluble PSP brain extract show an increase in the percentage of PSD95 puncta colocalizing with T22 when compared to slices cultured in medium or Tau −ve soluble PSP brain extract (Fig. 7b). Of the T22 positive staining in the slices treated with Tau +ve extract, 49.63% colocalized with PSD95 and 2.10% colocalized with GFAP-positive astrocytes, indicating that a substantial portion of the oligomeric tau taken up by the living human brain slice was internalized into postsynapses. There is also a trend for an increase in the percentage of PSD95 puncta colocalizing with AT8 in slices cultured with Tau +ve PSP brain extract, compared to slices cultured in medium alone or Tau −ve PSP extract (Fig. 7c); however, the effect size is smaller compared to T22, and over tenfold more postsynapses took up T22 than AT8-positive tau. Of the AT8-positive staining in the slices treated with Tau +ve extract, 46.16% colocalized with PSD95 and 0.45% colocalized with GFAP-positive astrocytes. These data suggest that PSP-derived tau can be taken up into living human postsynapses.

We then investigated the effects of PSP-derived tau on astrocytes in HBSCs. We found that there was a 3.4-fold increase in GFAP burden in slices cultured with Tau +ve PSP extract compared to medium alone and a 4.3-fold increase compared to Tau −ve PSP extract treatment (Fig. 7e), implying the astrogliosis observed is a result of the tau in PSP brain rather than any other factor. Furthermore, in the slices cultured with the Tau +ve PSP extract, there was an 11.6-fold increase in colocalization between PSD95 and GFAP compared to medium alone and a 14.2-fold increase compared to Tau −ve PSP extract (Fig. 7f). When the amount of GFAP and PSD95 colocalization was normalized to GFAP burden, there was no difference between treatments, suggesting astrogliosis drives the increased synaptic ingestion (Fig. 7g). Internalization of synapses by astrocytes was confirmed with immuno-gold electron microscopy (Fig. 7h–j).

## Discussion

In this study, we demonstrate that (1) tau accumulates within synaptic pairs in PSP, (2) pathological tau colocalizes with synaptogyrin-3 in presynapses, (3) astrocytes show increased synaptic engulfment in PSP, (4) clusterin is close enough to bind tau within human synapses and (5) PSP-derived tau can be taken up into postsynapses and induce astrogliosis and augmented synaptic engulfment in living HBSCs. Our experiments using a rare set of human postmortem brain samples prepared for array tomography and a new living human brain slice model^28,34^ challenged with PSP brain extract both support the idea that tau pathology spreads trans-synaptically in PSP. This is in line with lower-resolution human PET data showing that tau pathology in PSP accumulates in functionally connected brain circuits^18^ and with animal model data showing that human pathological tau spreads through brain regions via synaptic connections^11–16^. Recent data using array tomography and immunoelectron microscopy of human AD brain tissue similarly support the trans-synaptic spread of tau^10^^,^^36^. Additionally, the finding that tau on one side of the synapse strongly predicts the presence of tau on the other side indicates that tau could be spreading from neuron to neuron and brain region to brain region by moving through synaptic connections. While this does not establish a direct spatiotemporal sequence of tau spread, identifying this as a potential mechanism is important for future work aiming to slow or stop disease progression and synapse loss, which correlate with the spread of pathology.

Our finding that pathological tau accumulates inside synapses in PSP is also important, as this likely contributes to synapse loss in this disease. We observe that synaptic oligomeric tau correlates with synapse loss in PSP. This could be clinically important, as a monoclonal antibody that targets synaptic tau oligomers has entered a phase 1 clinical trial. A preprint suggests this antibody can block the synaptic uptake of oligomeric tau, promote neuronal survival and preserve synaptic integrity in a tauopathy mouse model^37^. We focused on excitatory synapses in this study as tau pathology preferentially affects excitatory neurons^38^. However, we have previously observed inhibitory synapse loss in AD^39^ and altered expression of genes important for inhibitory synapses in frontotemporal dementia due to a tau mutation^40^. Thus, in the future, it would be of interest to further characterize the types of synapses that contain tau in PSP and whether this corresponds with synapse-specific vulnerability.

Our proteomics data highlight changes in multiple synaptic proteins. In SN, we observe a loss of synaptic proteins, while in the frontal cortex, which is affected by pathology later, we see a reduction in some synaptic proteins but an increase in others, hinting at a potential compensatory response earlier in the disease process. Of particular interest, we observe increased levels of STX6 in the PSP frontal cortex. Genetic studies have identified a link between the *STX6* gene, which encodes STX6, and PSP risk^41,42^.

One way that tau is thought to induce synaptic loss and dysfunction is via its interactions with synaptic vesicles. Tau has been observed to interact with bassoon, a presynaptic scaffolding protein, and in both mouse and *Drosophila* models of tauopathy, bassoon was shown to exacerbate tau seeding and toxicity^43^. Furthermore, studies have demonstrated that tau and synaptogyrin-3 interact in mice and flies overexpressing human tau, causing clustering of synaptic vesicles, synapse loss and cognitive impairment^7,8,24^. Here we observed that in PSP, tau colocalizes in presynapses with synaptogyrin-3, and using electron microscopy, we observed oligomeric tau associated with synaptic vesicles. Our data, taken together with the previous animal data, indicate that in primary tauopathies as well as AD, tau-synaptogyrin-3 interactions may be important for synaptic dysfunction and loss.

Another potential mechanism of synapse loss in tauopathy involves glial engulfment of synapses. In AD, we recently observed that both microglia and astrocytes contain synaptic protein, with astrocytes engulfing more synapses than microglia^27^. Here we observed increased colocalization of synaptic proteins within astrocytes both in human postmortem PSP tissue and in our human brain slices challenged with PSP-derived tau, indicating that this synaptic ingestion by astrocytes is not an end-stage phenomenon of the neurodegenerative disease. In our living HBSCs experiments, immunodepleting tau from the PSP brain extract prevented astrogliosis and astrocyte engulfment of synapses, indicating that pathological tau in PSP brain specifically induces these phenotypes. Our proteomics results also indicate there are molecular changes in astrocytes in PSP, as we observe changes in levels of GFAP, YKL40/CHI3L1 and ICAM1 and several neuro-immune pathways.

Interestingly, we find no evidence for microglial activation or increased synaptic engulfment by microglia in PSP frontal cortex, in contrast with our previous finding in Alzheimer’s postmortem brain that there is an increase in colocalization between synaptic proteins and microglia^27^. However, in our recent study of another primary tauopathy, frontotemporal dementia due to the 10 + 16 mutation in the *MAPT* gene encoding tau, we observed no increases in synaptic ingestion by astrocytes or microglia^40^. These data indicate differences in synapse–glia interactions in different tauopathies. In accordance with our immunohistochemistry results, our proteomic analysis revealed no significant alterations in proteins commonly associated with microglial activation. A recent study with a larger sample size similarly found no significant difference between PSP and control frontal cortex (Brodmann area 9 (BA9)) when assessing microglial burdens^44^. Despite the lack of microglial changes, we do observe changes in complement cascade proteins in PSP in line with previous data implicating the complement pathway in disease pathogenesis^42^. This is of particular interest given the known role complement has in mediating synaptic pruning^25,45^.

Our proteomics data revealed increased clusterin protein levels in the frontal cortex in PSP. The clusterin gene *CLU* has been implicated in Alzheimer’s risk^46^, and we previously observed clusterin in synapses with amyloid β around plaques in Alzheimer’s brain^33^. Clusterin has been implicated in tau fibrillization in model systems and binding tau in NFTs in Alzheimer’s^47–49^, and a recent single-nucleus-sequencing study demonstrated increases in clusterin expression in hybrid astrocyte/oligodendrocyte cells in PSP brain^50^. To our knowledge, there were previously no studies examining clusterin and tau in synapses. We observe clusterin–tau colocalization in postsynapses, which may be a mechanism driving postsynaptic uptake of pathological tau in this region.

Due to the scarcity of PSP postmortem brain tissue suitably prepared for array tomography, our study is limited by our relatively small sample size and lack of power to observe any sex effects. It is also important to acknowledge that the living human brain slice tissue, although taken from areas not containing tumor, is from patients who have underlying tumor pathology. This may influence the cellular microenvironment, which could affect responses to challenges with PSP-derived tau. We mitigated this potential confound by treating adjacent slices from the same patient tissue sample with control, tau-immunodepleted or tau-containing PSP brain homogenates.

Despite the limitations of the experiments, our data are important as they provide evidence from both postmortem brain and living human brain slices that pathological tau accumulates in synapses, where it is associated with synapse loss and astrocytic engulfment of synapses. Based on our data, we conclude that tau pathology could spread at least in part via synapses in PSP, and we reveal potential mechanisms leading from pathological tau to synapse degeneration. These data suggest that targeting synaptic tau may attenuate disease progression in PSP and should be considered as part of multimodal therapy.

## Methods

### Human postmortem samples and ethical approval

Postmortem human brain tissue was acquired from the Edinburgh Brain Bank. The Edinburgh Brain Bank is a Medical Research Council-funded facility with research ethics committee approval (16/ES/0084). The details of the postmortem human cases (*n* = 11 PSP, 13 control) are given in Extended Data Table 1. PSP cases were included if they had a neuropathological diagnosis of PSP and a clinical diagnosis of a progressive neurodegenerative disorder. No statistical methods were used to determine sample sizes, as we were limited by the availability of tissue for this rare disease. Of our 11 PSP cases, 7 had a clinical diagnosis of PSP (Steele–Richardson–Olszewski, subtype unknown), 3 had clinical diagnoses of unknown neurodegenerative conditions and 1 had a diagnosis of Creutzfeldt–Jakob disease, which on pathology was found to be PSP. All 11 had neuropathologically confirmed PSP. Cases with significant copathologies were excluded. Control cases were matched for age (*t*-test, d*f* = 20.612, *t* = −1.51, *P* = 0.15), sex (*χ*^2^ test, d*f* = 1, *χ*^*2*^ = 0.021, *P* = 0.88) and postmortem interval (PMI; *t*-test, d*f* = 14.23, *t* = −2.12, *P* = 0.0516). Exclusion criteria for control cases included known clinical neurological or psychiatric disease or a neuropathological report consistent with a different neurodegenerative disorder. The array tomography study size was limited to seven per group by the available tissue embedded for array tomography. Simulation-based power analysis^51^ determined that an *n* = 7 would give a power = 0.78 to detect AT8 colocalization with SYO in PSP brain. Experiments were approved by the Edinburgh Brain Bank ethics committee, the Academic and Clinical Central Office for Research and Development (ACCORD) and the ACCORD medical research ethics committee (AMREC), a joint office of the University of Edinburgh and National Health Service Lothian (approval 15-HV-016). Donors did not receive compensation. All data have been pseudoanonymized, so personal identifying information cannot be accessed with these numbers. SN was chosen as a brain region that undergoes early accumulation of tau pathology, and the frontal cortex was chosen as a later-affected brain region where we postulate tau may be spreading to in late stages of disease. While there are other early and late-affected brain regions^2^, these were chosen due to the availability of tissue embedded for array tomography.

### Array tomography: tissue processing and immunohistochemistry

Samples from the frontal cortex (BA9) and SN were fixed and embedded for array tomography as described previously^52^. Briefly, fresh tissue samples were fixed in 4% paraformaldehyde, dehydrated in ethanol and embedded in London resin White. Samples were cut into ribbons of ultrathin serial 70 nm sections using a histo Jumbo Diamond knife (Diatome) and an Ultracut (Leica). Ribbons were rehydrated in 50 mM glycine and blocked in a solution of 0.1% fish skin gelatin and 0.05% Tween in tris-buffered saline for 45 min at room temperature. Ribbons were incubated with primary antibodies (Extended Data Table 2; including antibody dilutions) diluted in blocking solution overnight at 4 °C. Ribbons were washed with tris-buffered saline, and secondary antibodies (Extended Data Table 2) diluted in blocking solution were applied for 45 min at room temperature. When used, the direct label 488 SYO diluted in blocking solution (1:200) was applied for 1 h at room temperature after rinsing secondary antibodies off. Finally, ribbons were mounted on glass slides with ImmuMount. For more details, please see the methods video demonstrating this technique, as presented in ref. ^53^.

### Array tomography microscopy and image analysis

Images were obtained with a ×63/1.4 numerical aperture (NA) objective on an AxioImager (Zeiss; Zen software 3.0 blue edition) or Leica true confocal scanning (TCS) SP8 confocal (with software Leica application suite X 3.5.7.23225). Two regions of interest (ROIs) were chosen in the gray matter of BA9 or pars compacta SN and imaged in the same location on 15–30 sequential sections. Imaging parameters were kept the same throughout each experiment, and an image was taken of the negative control in each session to ensure there was no nonspecific staining. Image processing and analysis were performed blinded to case ID. Individual images from each ROI were combined into a 3D image stack, and a median background filter was applied in ImageJ. Using custom MATLAB scripts (MATLAB version v2022a), image stacks were aligned using rigid registration. Each channel was segmented using an auto-local thresholding algorithm to binarize images and remove any objects present in only a single section (from secondary antibody noise). Segmented images were run through custom MATLAB and Python scripts to determine object density in the neuropil and colocalization between channels. Objects were considered colocalized if at least 25% of the 3D volume overlapped. For presynaptic and postsynaptic objects to be considered a synaptic pair, the distance between the center of each object had to be ≤0.5 μm. 3D reconstructions were made with Imaris software (Bitplane). Saturation was minimized during image acquisition and only applied for figure visualization. All custom software scripts are available^54^.

### Immunohistochemistry on FFPE human tissue

Four micrometer-thick FFPE tissue sections from PSP and control frontal cortex and SN were provided by the Edinburgh Brain Bank. Sections were dewaxed with xylene (5 min) and rehydrated with decreasing concentrations of ethanol-to-water solutions. Samples were pressure-cooked for 3 min on the steam setting in citrate buffer (Vector Labs, H3300) for antigen retrieval. For immunofluorescence staining of astrocytes, microglia, tau and synapses, slides were then incubated with the autofluorescence eliminator reagent (Merck Millipore, 2160) and the Vector TrueVIEW Autofluorescence Quenching Kit (SP-8400-15) to reduce autofluorescence caused by lipofuscin and red blood cells. Slides were outlined using a PAP pen (Daido Sangyo, 1-5902-12) and incubated with blocking solution for 1 h at room temperature (PBS with 0.3% Triton X-100 and 10% normal donkey serum (Sigma-Aldrich, D96663)) and then incubated at 4 °C overnight with primary antibodies (Extended Data Table 2). Sections were washed and incubated with secondary antibodies at room temperature for 2 h (Extended Data Table 2). If used, the directly labeled anti-SYO antibody was applied at room temperature for 45 min. Slides were mounted onto glass coverslips using ImmuMount. Images were acquired using a confocal microscope (Leica, TCS8) with a ×63 oil immersion objective. Ten image stacks were taken for each case, and the brain region was sampled randomly through all six cortical layers. Laser and detector settings were kept constant. Using a custom MATLAB script, the image stacks were segmented, and the volume of the image stack occupied by each channel and the colocalization between channels were calculated. To calculate tau burdens, following dewaxing and rehydration, slides were labeled with AT8 antibody recognizing tau phosphorylated at ser202/thr205 and visualized using the Leica Novolink polymer detection kit (RE7280-CE) as per the manufacturer’s instructions. To calculate the tau burdens, stereology was undertaken on the AT8-stained slides using Zeiss Axioimager Z2, and raw burden data were calculated with Stereoinvestigator (MicroBrightField, v.2022.3.1) and Neurolucida Explorer software (v.2021.1.1) by obtaining values for total area and total area of AT8 stain detected. Stereoinvestigator software requires manual selection of positive staining for autodetection of the total area with staining; therefore, cases with no visible staining were manually assigned a zero value. The experimenter was blinded to case information during imaging and analysis.

### FRET

FRET images were taken to detect interactions between fluorophores <10 nm apart. Array tomography ribbons were immunostained to examine if postsynapses (PSD95) and clusterin colocalize and if there is a positive FRET signal between postsynapses (PSD95) and oligomeric tau (T22) and between oligomeric tau (T22) and clusterin within postsynapses. Images of sequential array tomography sections of the same field of view were taken using a Leica TCS8 confocal (Leica application suite X 3.5.7.23225 software) with a ×63/1.4 NA oil objective. For FRET analysis, the signal intensity in the acceptor (594 or 647) channel was measured when only the donor laser (488 or 552 excitation wavelength) was exciting the sample, thus recording the energy transfer from donor molecules to acceptors based on intensity (emission FRET). Laser and detector settings were set at the beginning of each imaging session on a positive control to avoid saturation. For FRET analysis, donor-only (488 or 594) and acceptor-only (594 or 647) samples were prepared (Extended Data Table 3) and imaged to calculate the donor emission cross-talk with the acceptor emission and the direct excitation of the acceptor by the donor excitation laser line^55^. Positive control samples labeling the same protein with both donor and acceptor fluorophores with secondary and tertiary antibodies were stained and imaged to ensure FRET signals could be detected in each experiment. Images were aligned and thresholded using in-house Fiji (ImageJ) and MATLAB scripts, following the standard array tomography procedure described above. Stacks of aligned images corresponding to the acceptor emission under donor excitation (referred to as the FRET image) were corrected for donor- and acceptor-only confounds. Thresholded image masks corresponding to the donor and the acceptor images were used to identify areas of overlap in the corrected FRET image. These areas were used to quantify the percentage of pixels exhibiting any FRET signal, providing a qualitative measure of the presence of the FRET effect.

### Soluble protein extraction from PSP brain and tau immunodepletion

In total, 5.3 g of PSP frontal cortex (BA6/BA8) was thawed on ice, finely diced and homogenized in 30 ml of 1× artificial cerebrospinal fluid (aCSF, pH 7.4) supplemented with three complete mini EDTA-free protease inhibitor cocktail tablets (Roche, 11836170001) using a Dounce homogenizer. Homogenized tissue was transferred to 15 ml protein LoBind tubes (Eppendorf, 0030122216), placed on a roller for 30 min at 4 °C and then centrifuged at 2,000*g* at 4 °C for 10 min to remove insoluble debris. The supernatant was centrifuged again for 110 min at 200,000*g* and 4 °C. The resulting supernatant, which here we will call the soluble extract, was dialyzed in aCSF for 72 h (Slide-A-Lyzer G2 Dialysis Cassettes) to remove salts, impurities and drugs the donor took. The aCSF was replaced every 24 h. Following dialysis, the extract was pooled and divided into two. The two portions of extract were incubated with protein A agarose (PrA) beads (30 μl ml^−1^ of sample; Thermo Fisher Scientific, 20334) and either a total tau antibody Tau13 (mouse IgG1; BioLegend, MMs-520R) to create the Tau −ve extract or an anti-GFP antibody (mouse IgG1, 1:50, DSHB, DSHB-GFP-8H11) to create the Tau +ve extract, overnight at 4 °C on a rotating mixer. Then the solutions were centrifuged at 2,500*g* for 5 min, the supernatant was collected into fresh protein LoBind tubes and incubated again with a fresh PrA beads/antibody solution for a total of four times. Finally, the Tau +ve and Tau −ve supernatant were collected into fresh protein tubes and stored at −80 °C. The concentrations of tau in each of the extracts were quantified by sandwich enzyme-linked immunoassay according to the manufacturer’s instructions (Thermo Fisher Scientific, KHB0041; Thermo Scientific Xcalibur v.4.5.474.0). The total tau concentration in the Tau −ve extract = 0.013 μg ml^−1^ and in the Tau +ve extract = 11.14 μg ml^−1^.

### Tissue and ethical approval for living HBSCs

Use of living resected human tissue has been approved by AMREC and Lothian NRS Bioresource (Spires-Jones and Durrant; REC, 15/ES/0094; IRAS, 165488; Bioresource, SR1319). Additional Caldicott Guardian approval (CRD19080) has been obtained to receive data about the sex, age (in years), brain region the tissue was taken from and the clinical reason for surgery. The informed consent of patients was obtained using the Lothian NRS Bioresource Consent Form; no compensation or incentive was offered to patients to consent to donating surplus tissue. Case information for the patients used in this study is outlined in Extended Data Table 1.

### Preparation and maintenance of HBSCs

HBSCs were made using surplus, peritumoral cortical access tissue surgically resected from patients undergoing tumor debulking surgery as previously described^28^^,^^56^^,^^37^. Tissue was collected at surgery into ice-cold, 0.22 μm-filtered, and continuously oxygenated aCSF containing 87 mM NaCl, 2.5 mM KCl, 10 mM HEPES, 1.62 mM NaH_2_PO_4_, 25 mM d-glucose, 129.3 mM sucrose, 1 mM Na-pyruvate, 1 mM ascorbic acid, 7 mM MgCl_2_ and 0.5 mM CaCl_2_. The peritumoral cortical tissue was then embedded in 4% agar and sectioned using a Leica VT1200S vibratome into 300-µm-thick sections. The tissue was further dissected so that each slice contained all the cortical layers and a small amount of white matter for orientation. Slices were then placed into a wash buffer composed of continuously oxygenated Hanks balanced salt solution and HEPES (20 mM; 305 mOsm, pH 7.3) for 15 min at room temperature and then plated on 0.4-μm pore membranes (Millipore, PICM0RG50) sitting on top of 750 μl of a second wash solution (BrainPhys Neuronal Culture Medium (StemCell Technologies) (96%), N2 (1×), B27 (1×), hBDNF (40 ng ml^−1^), hGDNF (30 ng ml^−1^), Wnt7a (30 ng ml^−1^), ascorbic acid (200 nM), dibutyryl cAMP (1 mM), laminin (1 µg ml^−1^), penicillin–streptomycin (1%), nystatin (3 U ml^−1^) and HEPES (20 mM)). Slice cultures were kept in the second wash medium in an incubator at 37 °C with 5% CO_2_ for 1–2 h, after which the medium was aspirated, and slices were randomly allocated to treatment with one of three different solutions. Medium condition slices were cultured in 100% maintenance medium (same as ‘second wash solution’ mentioned above, but without HEPES). Tau −ve condition slices were cultured in 75% maintenance medium and 25% Tau −ve PSP brain extract (final total tau concentration <0.003 μg ml^−1^). Tau +ve condition slices were cultured in 75% maintenance medium and 25% Tau +ve PSP brain extract (final total tau concentration = 2.789 μg ml^−1^). The aCSF, wash solutions and maintenance medium were passed through a 0.22 μm filter before use to ensure sterility. To prevent the removal of protein aggregates, the PSP extracts were not filtered, but we did not encounter contamination issues. Cultures were inspected daily for signs of bacterial, fungal or yeast infections. After 72 h, the slices were processed for array tomography and immunostained as detailed above. The antibody information is listed in Extended Data Table 2. Our living human brain slices were generated from surgically resected tissue from temporal (*n* = 2), frontal (n = 3) or parietal/occipital cortex (*n* = 1), depending on tumor location. The uptake of tau by synapses was not noticeably different by brain region in these experiments.

### *APOE* genotyping

Samples were genotyped by the Edinburgh Genetics Core. All samples were genotyped using the TaqMan SNP Genotyping Assays. Taqman genotyping was carried out on the QuantStudio12KFlex to establish *APOE* variants using the following assays: C___3084793_10 for rs429358 and C____904973_10 for rs7412.

### Proteomics

Total homogenates (TH) and biochemically isolated synaptic enriched fractions were prepared for proteomics as previously published^57^. Briefly, 300–500 mg of PSP or control frontal cortex or SN was homogenized using a Dounce homogenizer with 1 ml of buffer (25 mM HEPES, 120 mM NaCl, 5 mM KCl, 1 mM MgCl_2_, 2 mM CaCl_2_ and protease inhibitors (Merck Millipore, 11836170001) and phosphatase inhibitors (Merck Millipore, 524629-1SET), made up in sterile water). Homogenate was aspirated in a 1 ml syringe and passed through a presoaked 80 μm filter (Merck Millipore, NY8002500) to remove debris and yielded the TH. A portion of the TH sample was passed through a presoaked 5-μm filter (Merck Millipore, SLSV025LS) followed by centrifugation at 2,000*g* for 5 min to yield the synaptic enriched fraction pellet. The supernatant was discarded, and the pellet was washed with buffer and centrifuged a second time at 2,000*g* for 5 min to ensure purity.

Samples were diluted fivefold with Tris–HCl buffer (pH 7.6; 100 mM Tris–HCl, 4% SDS and protease inhibitor cocktail EDTA-free (Thermo Fisher Scientific, 78447)) and homogenized thoroughly with a pipette tip. The samples were centrifuged for 20 min at 17,000*g* at 4 °C. The supernatant was aliquoted into fresh protein LoBind Eppendorf’s and stored at −80 °C. Micro BCA Protein Assay Kit (Thermo Fisher Scientific, 23235) was used to quantify protein levels, following the manufacturer’s instructions. Absorbance values were obtained using spectrophotometry at 562 nm, with the working solution used as the blank value to calibrate the machine.

Peptide samples were resuspended in 0.1% formic acid before loading into vials for mass spectrometry (MS) analysis. Samples were acquired on a Thermo Exploris 480 (software Thermo Tune for the Exploris 480 v.4.1.335.19) connected in line with an Ultimate 3000 UPLC (Thermo Fisher Scientific). In total, 1 µg of peptides was loaded in 1 µl onto a 5 µm, 100 µm × 2 cm nanoViper C18 trap column (Thermo Fisher Scientific). Peptides were separated using a 2 µm, 75 µm × 50 cm C18 reversed-phase easy-spray analytical column over 135 min at a flow rate of 300 nl min^−1^. A linear gradient from 3% to 35% was used from water with 0.1% formic acid to acetonitrile with 0.1% formic acid. MS data was acquired in data-independent mode using a 45 variable *m*/*z* window method.

**Extended Data Fig. 7:**
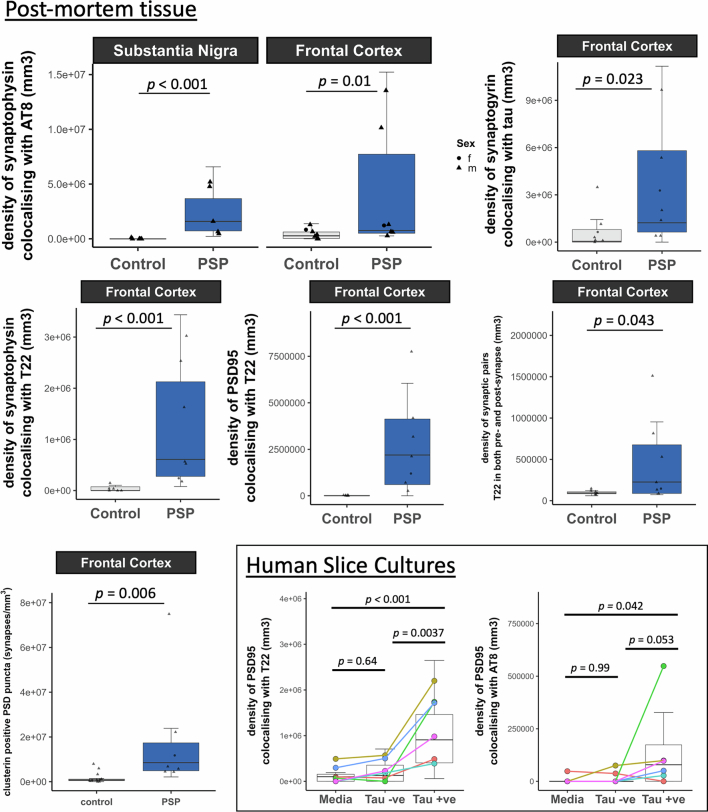
Alternative analyses assessing differences in density of synaptic colocalization. Array tomography data presented in the main figures as the percentage of synapses containing tau are shown here as the density of tau-containing synapses, showing that on the order of 10^6^ synapses per mm^3^ contain tau in both post-mortem brain and living human brain slice cultures challenged with PSP-derived tau. Synpatophysin-AT8 post-mortem data are from (n = 5 donors substantia nigra, 7 frontal cortex) compared to control (n = 6 substantia nigra, 7 frontal cortex). Two image stacks per region per case were taken as technical replicates. Density of AT8-positive synaptophysin puncta is significantly higher in PSP (ANOVA after linear mixed effects model on Tukey transformed data, F(10.7) = 22.864, p = 0.0006). Post hoc tests show increases in AT8-positive synaptophysin density in both substantia nigra (estimate = 47.3, 95% CI (27.93, 66.6), *t*(21.1) = 5.081, *p* < 0.0001) and frontal cortex (estimate = 27.3, 95% CI (9.33, 45.2), *t*(17) = 3.208, *p* = 0.005). Quantifications of synaptogyrin and T22 data are from the frontal cortex of n = 7 PSP, 7 control cases with 2 image stacks per case as technical replicates analyzed with a linear mixed effects model of Tukey-transformed data ~ diagnosis + 1 | case with post hoc Tukey-corrected tests. There is an increase in PSP in the density of synaptophsin objects colocalized with T22 (estimate = 70.2, 95% CI (37.7, 103), *t*(10.7) = 4.771, *p* = 0.0006), PSD95 puncta with T22 (estimate = 143, 95% CI (80.5, 206), *t*(10.6) = 5.047, *p* = 0.0004) and density of synaptic pairs with T22 in both pre and post (estimate = 3.84 × 10^−7^, 95% CI (9.44 × 10^−9^, 7.59 × 10^−7^), *t*(10.3) = 2.276, *p* = 0.043). Human slice cultures are from n = 6 human brain tissue donors, each treated with three conditions including two stacks (technical replicates) per condition. There is a significant effect of treatment on the density of objects consistent with T22 (ANOVA after linear mixed effects model on Tukey transformed data ~ treatment + 1 | donor, *F*(31.017) = 16.974, *p* < 0.00001) and AT8 (*F*(14.328) = 5.8542, *p* = 0.0247). Boxes show quartiles and medians calculated from each image stack; whiskers show minima and maxima excluding outliers. Data points show case means (biological replicates).

MS.raw files were processed using DIA-NN (v.1.8.1)^58^ using the ‘library free’ method. A human UniProt FASTA (downloaded on 1 September 2023) was used as the reference database for library generation. The default settings were used except for heuristic protein inference. Protein inference was set to ‘protein names’, and the double-pass mode for the neural network classifier was used. Resultant protein abundance matrices were then processed using R (v.4.1.3). Protein abundance values were normalized using the quantile normalization as part of the PreProcessCore package. Missing values were imputed using the ImpSeqRob function as part of the rrcovNA package using the default parameters. Statistical comparisons were then performed using the Limma package. Comparisons were made using the ‘eBayes’ method. DEPs were defined as proteins that exhibited more than a 20% alteration in protein levels (fold change <0.8 or >1.2) and a *P* value < 0.05. The choice of a 20% change as a threshold was motivated by the consideration that such alterations likely represent biologically meaningful shifts in protein levels. DEPs were analyzed using GO enrichment (‘clusterProfiler’ R package)^35^ and the synaptic GO tool, SynGO^59^, with a *P* value cutoff = 0.05, *q* value cutoff = 0.05 and *P* value adjustment method set to Benjamini–Hochberg correction for multiple comparisons. To ensure that differences in the percentage of putative synapses colocalizing with pathology throughout this study were not solely due to synapse loss, we also assessed the density of putative synapses with pathology (Extended Data Fig. 7).

### Western blot

After protein extraction, each sample was made up to 15 μg of protein in 15 μl of deionized water as calculated by the micro BCA and diluted in half with Laemmli buffer (2× stock; S3401-10VL). In each well, 15 μl of sample was loaded in 4–12% Bis–Tris gels (Thermo Fisher Scientific, NP0323BOX). Each gel was run with 5 μl of molecular weight marker (LI-COR, 928-40000). Gels were run at 80 V for 5 min, followed by 120 V for 1.5 h. Gels were washed in 20% ethanol for 10 min before transferring using the iBlot 2 Dry Blotting System IB21001 as per the manufacturer’s instructions. Prepacked transfer stacks containing a PVDF membrane (Thermo Fisher Scientific, IB24002) were assembled, and samples were transferred for 8.5 min at 25 V. After transferring, we used the LI-COR Revert 700 total protein stain kit (LI-COR Biosciences, 926-11010) for western blot normalization, as per the manufacturer’s instructions. Membranes were then blocked for 1 h using PBS intercept blocking buffer (LI-COR Biosciences, 927-70001). Primary antibodies were diluted in PBS intercept blocking buffer with 0.1% Tween-20 and incubated with membranes overnight at room temperature. Membranes were washed three times for 5 min with PBS–Tween and then incubated in darkness for 2 h with secondary antibodies IRDye 800CW donkey anti-rabbit (LI-COR Biosciences, 925-32213) and IRDye 680RD donkey anti-mouse (LI-COR Biosciences, 925-680RD), both at 1:5,000 concentration. Membranes were washed 3× in PBS–Tween, 1× in PBS and then imaged using a LI-COR Odyssey Fc machine (software LI-COR Image Studio v 5.2). Synaptic enriched fraction preparations were assessed for enrichment for SYO or PSD95 and exclusion of histones (Extended Data Fig. 4a–c). Preparations that did not show enrichment for synaptic proteins were repeated.

### Electron microscopy

For immunogold electron microscopy, tissue was processed and embedded in resin blocks as described above for array tomography. The resin-embedded tissue blocks were cut into 50 nm ultrathin sections using an ultramicrotome (Leica, UC6) fitted with a Histo Jumbo Diamond Knife (Diatome Knives) onto Formvar-coated nickel grids (Agar Scientific, S162N1). Alternatively, to examine the species of tau present in the Tau +ve PSP brain extract, 5 µl of extract was pipetted onto the Formvar-coated grids and allowed to bind for 1 min before removing excess sample with filter paper. Grids were incubated in 1% sodium borohydride in 0.1 M PB for 5 min to reduce free aldehydes and thereafter incubated in 50 mM glycine in 0.1 M PB for 10 min to inactivate residual aldehyde groups. The grids were then blocked in 0.1% BSA, 0.1% fish skin gelatin and 0.05% Tween in 0.2 M PB (pH 7.4) for 1 h at room temperature. Primary antibodies (Extended Data Table 2) were diluted in antibody dilution buffer (0.1% BSA and 150 mM sodium chloride in 0.2 M PB (pH 7.4)) and incubated with the nickel grids overnight at 4 °C. The grids were washed six times in 0.2 M PB, and 10 nm gold-conjugated secondary antibodies (Extended Data Table 2) were diluted in antibody dilution buffer and incubated with the nickel grids for 90 min at room temperature. The nickel grids were then washed six times in 0.2 M PB, fixed in 2.5% glutaraldehyde in 0.2 M PB for 15 min, washed six times in 0.2 M PB, treated with 0.1% osmium tetroxide for 20 min and finally rinsed with ddH_2_O three times for 5 min each wash. Negative staining was carried out using 3% uranyl acetate in 50% ethanol for 15 min and 3% lead citrate in ddH_2_O for 150 s. Images were captured using a transmission electron microscope (JEM-1400 Plus, JOEL).

Synaptic enriched fraction pellets were fixed in 4% paraformaldehyde, 2.5% glutaraldehyde and 0.2% picric acid in 0.1 M phosphate buffer (pH 7.4) for 2 h at room temperature plus overnight at 4 °C, postfixed in 1% osmium tetroxide for 30 min, washed in 0.1 M PB, boiled distilled water and 50% ethanol, incubated in 1% uranyl acetate in 70% ethanol, dehydrated through 15 min steps in a graded series of ethanol then propylene oxide, 50% propylene oxide/50% Durcupan resin, then 100% Durcupan resin overnight in a Leica EM TP processor. Samples were baked in Durcupan resin in agar capsules overnight at 56 °C and cut on an Ultracut microtome (Leica) with a Histo Jumbo diamond knife (Diatome) into 50 nm sections, which were mounted on nickel mesh grids. Electron micrographs were captured on a Zeiss Gemini 360 scanning electron microscope with an annular STEM detector.

### Statistical analyses

Statistical analysis was performed using RStudio with R v.4.3.1. No data points were excluded from the analyses. The analysis and visualization of the data were facilitated by using the large language model ChatGPT, which was used to assist in refining R code. The majority of statistical analyses used linear mixed effects models (LMEMs; ‘lme4’ R package v.1.1-35.3), as this allowed us to test if diagnosis or treatment group impacted our variable of interest while controlling for potentially confounding variables, such as age and PMI, and including random effects to account for repeated measures. The decision to ultimately include potentially confounding fixed and random effects in the model and prevent overfitting of the model was made by inspecting the Akaike information criterion and Bayesian information criterion to assess which model best fits the data. LMEMs assume linearity, normal distribution of residuals and homogeneity of variance. Linearity was assessed by plotting model residuals against predictors, normality of residuals was checked with a QQ plot and homogeneity of variance was checked by plotting residuals against fitted values. If the model did not meet the assumptions, data were transformed using the method that best transformed each individual model to fit the assumptions. Data transformations included square root, log, arcsine square root and Tukey transformation. Post hoc testing was conducted for pairwise comparisons, estimated marginal means and 95% confidence intervals (CIs; ‘emmeans’ package), with *P* values adjusted using Tukey correction for multiple comparisons. Effect sizes are displayed as differences in estimated marginal means (‘emmeans’ package) and displayed as ‘estimate’ with 95% CIs for the effect size. When data are transformed to meet the model assumptions, then the reported effect sizes are computed on the transformed data. Degrees of freedom were calculated using the Kenward–Roger approximation. Correlations used Pearson’s or Spearman’s methods, dependent on whether the data met the assumptions for parametric testing or not. Full statistical analysis code for all figures are shared in ref. ^60^.

### Reporting summary

Further information on research design is available in the Nature Portfolio Reporting Summary linked to this article.

## Online content

Any methods, additional references, Nature Portfolio reporting summaries, source data, extended data, supplementary information, acknowledgements, peer review information; details of author contributions and competing interests; and statements of data and code availability are available at 10.1038/s41593-025-01992-5.

## Supplementary information

Reporting Summary

## Figures and Tables

**Extended Data Table 1:**
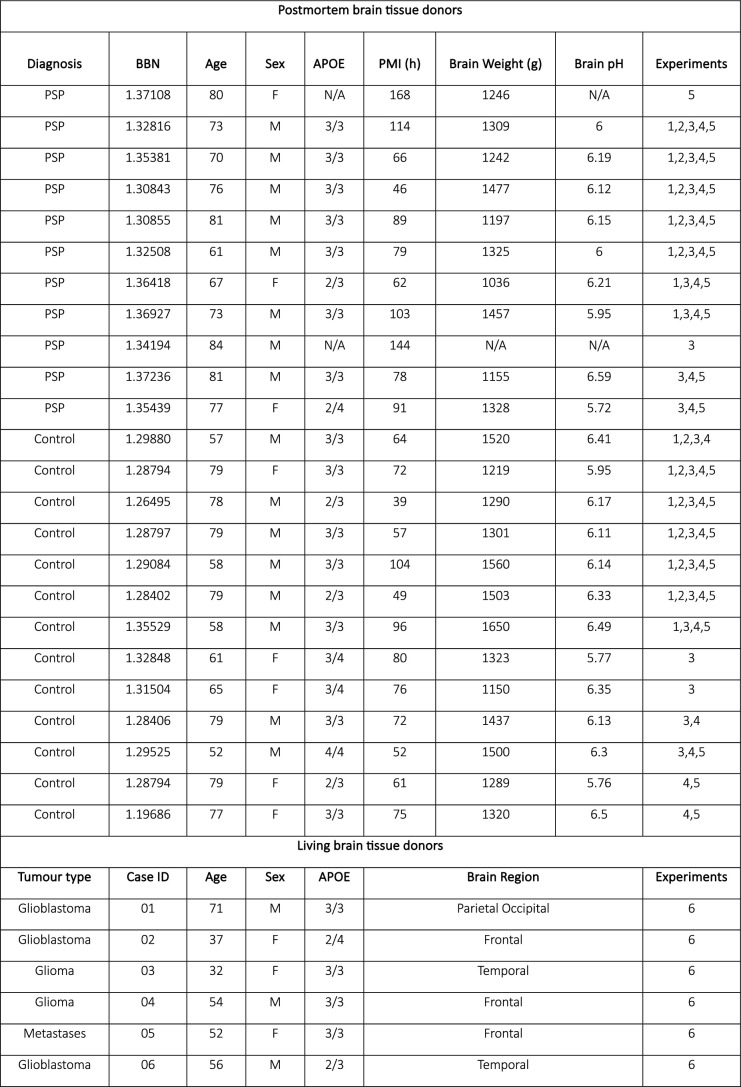
Details of brain tissue donors

1, array tomography on frontal cortex; 2, array tomography on substantia nigra; 3, proteomics analysis of frontal cortex; 4, proteomics analysis of substantia nigra; 5, analysis of FFPE tissue for evidence of synaptic engulfment by glia; 6, living organotypic brain slices. BBN, UK Brain Bank Number. Cases with APOE status listed as N/A did not have consent for genetic testing.

**Extended Data Table 2:**
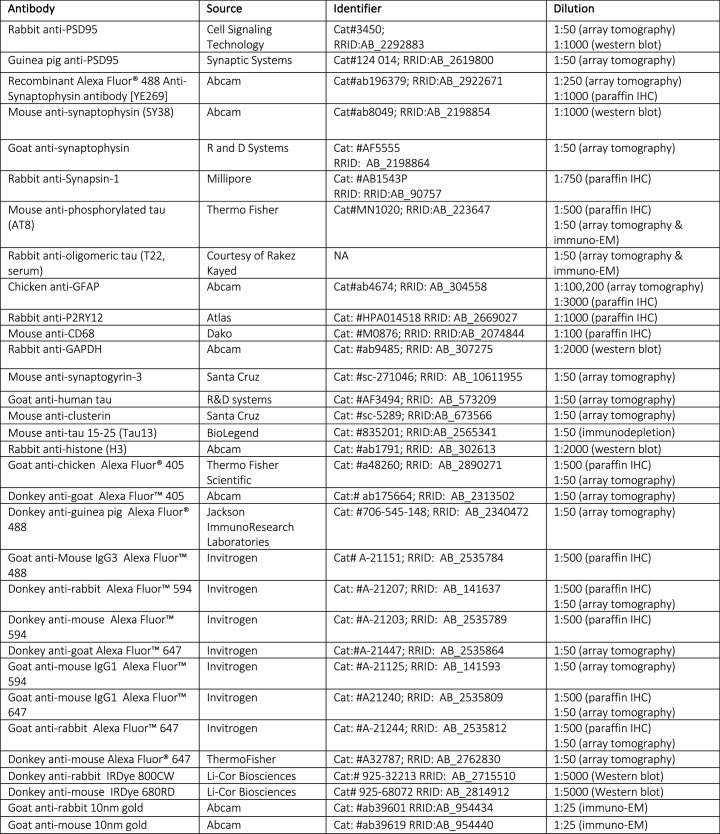
Antibody information

IHC, immunohistochemistry; immuno-EM, immunogold electron microscopy.

**Extended Data Table 3:**
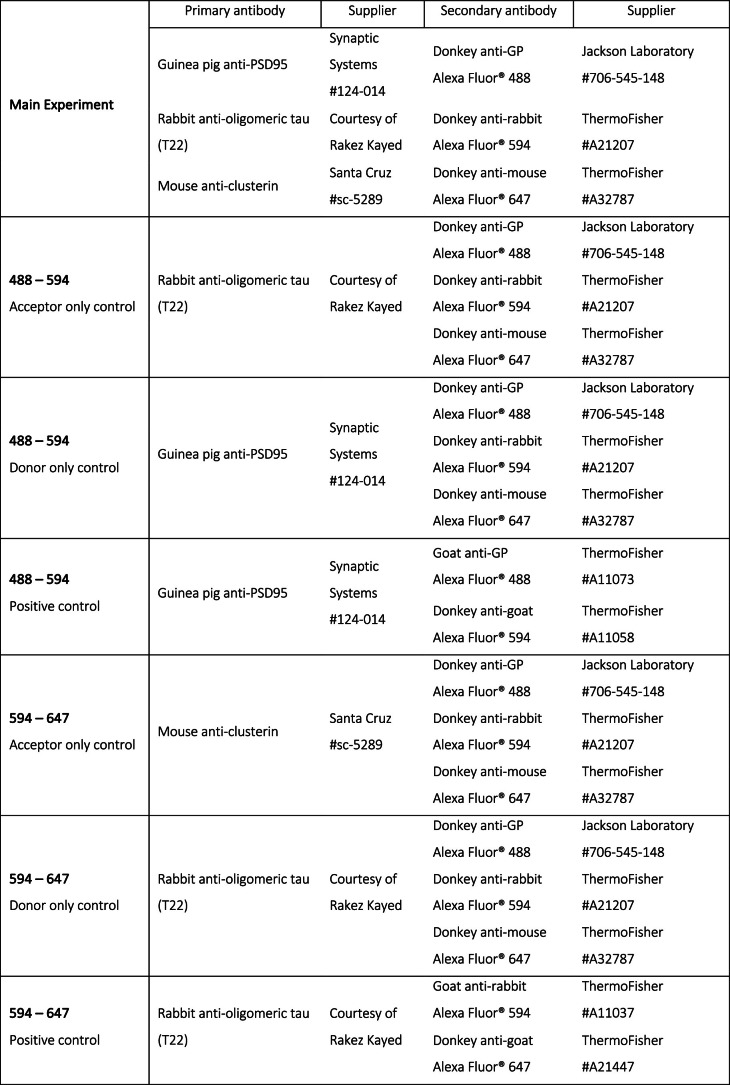
Table summarizing the antibody combinations used in FRET experiments—all dilutions 1:50

## Data Availability

Data and statistical analysis files are available on Edinburgh DataShare^60^. Proteomics data are available through the PRIDE repository datasets PXD047282 and PXD048456. Raw images are available from the lead authors upon request.

## Code availability

Custom analysis code for array tomography is freely available online^54^.
