# Supplementary material for: Evidence for trans-synaptic propagation of oligomeric tau in human progressive supranuclear palsy
Source: Nat Neurosci. 2025 Jul 16;28(8):1622–34. doi: 10.1038/s41593-025-01992-5 (PMC12321572; doi:10.1038/s41593-025-01992-5)
Supplement: Supplementary file 1 — Reporting Summary [file 41593_2025_1992_MOESM1_ESM.pdf]

nature portfolio

Corresponding author(s): Spires-Jones and Durrant

Last updated by author(s): Apr 8, 2025

Reporting Summary

Nature Portfolio wishes to improve the reproducibility of the work that we publish. This form provides structure for consistency and transparency in reporting. For further information on Nature Portfolio policies, see our Editorial Policies and the Editorial Policy Checklist.

Statistics

For all statistical analyses, confirm that the following items are present in the figure legend, table legend, main text, or Methods section.

n/a	Confirmed	
☐	☒	The exact sample size (*n*) for each experimental group/condition, given as a discrete number and unit of measurement
☐	☒	A statement on whether measurements were taken from distinct samples or whether the same sample was measured repeatedly
☐	☒	The statistical test(s) used AND whether they are one- or two-sided<br>*Only common tests should be described solely by name; describe more complex techniques in the Methods section.*
☐	☒	A description of all covariates tested
☐	☒	A description of any assumptions or corrections, such as tests of normality and adjustment for multiple comparisons
☐	☒	A full description of the statistical parameters including central tendency (e.g. means) or other basic estimates (e.g. regression coefficient) AND variation (e.g. standard deviation) or associated estimates of uncertainty (e.g. confidence intervals)
☐	☒	For null hypothesis testing, the test statistic (e.g. *F*, *t*, *r*) with confidence intervals, effect sizes, degrees of freedom and *P* value noted<br>*Give P values as exact values whenever suitable.*
☒	☐	For Bayesian analysis, information on the choice of priors and Markov chain Monte Carlo settings
☐	☒	For hierarchical and complex designs, identification of the appropriate level for tests and full reporting of outcomes
☐	☒	Estimates of effect sizes (e.g. Cohen's *d*, Pearson's *r*), indicating how they were calculated

Our web collection on statistics for biologists contains articles on many of the points above.

Software and code

Policy information about availability of computer code

Data collection	Zeiss Zen software 3.0 (blue edition); Leica application suite X 3.5.7.23225; Licor Image Studio v 5.2; Themo Scientific Xcalibur v.4.5.474.0; Thermo Tune for the Exploris 480 v. 4.1.335.19; Stereo investigator (MicroBrightField, version 2022.3.1); Neurolucida Explorer software (version<br>2021.1.1)
Data analysis	DIA-NN version 1.8.1, R (version 4.1.3), R lme4 package (v 1.1-35.3) MATLAB v2022a, custom scripts shared here: https://doi.org/10.7488/ds/7875

For manuscripts utilizing custom algorithms or software that are central to the research but not yet described in published literature, software must be made available to editors and reviewers. We strongly encourage code deposition in a community repository (e.g. GitHub). See the Nature Portfolio guidelines for submitting code & software for further information.

Data

Policy information about availability of data

All manuscripts must include a data availability statement. This statement should provide the following information, where applicable:

- Accession codes, unique identifiers, or web links for publicly available datasets
- A description of any restrictions on data availability
- For clinical datasets or third party data, please ensure that the statement adheres to our policy

Data and statistical analysis files are available on Edinburgh Datashare https://doi.org/10.7488/ds/7908. Proteomics data are available through the PRIDE repository datasets PXD047282 and PXD04845

Research involving human participants, their data, or biological material

Policy information about studies with human participants or human data. See also policy information about sex, gender (identity/presentation), and sexual orientation and race, ethnicity and racism.

Reporting on sex and gender	Sex of all donors is included in tables.
Reporting on race, ethnicity, or other socially relevant groupings	We do not have data on gender or race.
Population characteristics	Demographic data for each participant including age is included in the extended data table 1. Groups were matched for age and sex.
Recruitment	Tissue was collected from tissue banks and people undergoing brain surgery. There are likely biases in self-selection for participation in research which we did not have control over.
Ethics oversight	Edinburgh Brain Bank ethics committee, the Academic and Clinical Central Office for Research and Development (ACCORD) and the medical research ethics committee AM REC a joint office of the University of Edinburgh and National Health Service Lothian, Caldicott Guardian

Note that full information on the approval of the study protocol must also be provided in the manuscript.

Field-specific reporting

Please select the one below that is the best fit for your research. If you are not sure, read the appropriate sections before making your selection.

☒ Life sciences ☐ Behavioural & social sciences ☐ Ecological, evolutionary & environmental sciences

For a reference copy of the document with all sections, see nature.com/documents/nr-reporting-summary-flat.pdf

Life sciences study design

All studies must disclose on these points even when the disclosure is negative.

Sample size	Sample sizes were determined by availability of tissue. Post-hoc power calculations were performed as in methods showing we have power of 0.78 to detect our main outcome measure in the study with available sampes
Data exclusions	No data were excluded from the analysis
Replication	We have internal replication with two markers of tau in synapses which show consistent results (AT8 and T22) and replicate the main finding that postsynapses can take up tau in a live human brain slice model system with both AT8 and T22.
Randomization	human brain slice dishes were randomly allocated to treatment groups and slices from each donor received all 3 treatments
Blinding	Experimenters were blinded to case and treatment information during data analysis. Experimenters did not know case information during data collection but tau pathology was often evident so it was impossible to be truly blinded at this stage.

Reporting for specific materials, systems and methods

We require information from authors about some types of materials, experimental systems and methods used in many studies. Here, indicate whether each material, system or method listed is relevant to your study. If you are not sure if a list item applies to your research, read the appropriate section before selecting a response.

Materials & experimental systems

n/a	Involved in the study
☐	☒ Antibodies
☒	☐ Eukaryotic cell lines
☒	☐ Palaeontology and archaeology
☒	☐ Animals and other organisms
☒	☐ Clinical data
☒	☐ Dual use research of concern
☒	☐ Plants

Methods

n/a	Involved in the study
☒	☐ ChIP-seq
☒	☐ Flow cytometry
☒	☐ MRI-based neuroimaging

Antibodies

Antibodies used

Antibody Source Identifier Dilution
Rabbit anti-PSD95 Cell Signaling Technology Cat#3450; RRID:AB_2292883 1:50 (array tomography); 1:1000 (western blot)
Guinea pig anti-PSD95 Synaptic Systems Cat#124 014; RRID:AB_2619800 1:50 (array tomography)
Recombinant Alexa Fluor® 488 Anti-Synaptophysin antibody [YE269] Abcam Cat#ab196379; RRID:AB_2922671 1:250 (array tomography) 1:1000 (paraffin IHC)
Mouse anti-synaptophysin (SY38) Abcam Cat#ab8049; RRID:AB_2198854 1:1000 (western blot)
Goat anti-synaptophysin R and D Systems Cat: #AF5555 RRID: AB_2198864 1:50 (array tomography)

Rabbit anti-Synapsin-1 Millipore Cat: #AB1543P
RRID: RRID:AB_90757 1:750 (paraffin IHC)
Mouse anti-phosphorylated tau (AT8) Thermo Fisher Cat#MN1020; RRID:AB_223647 1:500 (paraffin IHC)
1:50 (array tomography & immuno-EM)
Rabbit anti-oligomeric tau (T22, serum) Courtesy of Rakez Kayed NA 1:50 (array tomography & immuno-EM)
Chicken anti-GFAP Abcam Cat#ab4674; RRID: AB_304558 1:100,200 (array tomography)
1:3000 (paraffin IHC)
Rabbit anti-P2RY12 Atlas Cat: #HPA014518 RRID: AB_2669027 1:1000 (paraffin IHC)
Mouse anti-CD68 Dako Cat: #M0876; RRID: RRID:AB_2074844 1:100 (paraffin IHC)
Rabbit anti-GAPDH Abcam Cat: #ab9485; RRID: AB_307275 1:2000 (western blot)
Mouse anti-synaptogyrin-3 Santa Cruz Cat: #sc-271046; RRID: AB_10611955 1:50 (array tomography)
Goat anti-human tau R&D systems Cat: #AF3494; RRID: AB_573209 1:50 (array tomography)
Mouse anti-clusterin Santa Cruz Cat: #sc-5289; RRID:AB_673566 1:50 (array tomography)
Mouse anti-tau 15-25 (Tau13) BioLegend Cat: #835201; RRID:AB_2565341 1:50 (immunodepletion)
Rabbit anti-histone (H3) Abcam Cat: #ab1791; RRID: AB_302613 1:2000 (western blot)
Goat anti-chicken Alexa Fluor® 405 Thermo Fisher Scientific Cat: #a48260; RRID: AB_2890271 1:500 (paraffin IHC)
1:50 (array tomography)
Donkey anti-goat Alexa Fluor™ 405 Abcam Cat:# ab175664; RRID: AB_2313502 1:50 (array tomography)
Donkey anti-guinea pig Alexa Fluor® 488 Jackson ImmunoResearch Laboratories Cat: #706-545-148; RRID: AB_2340472 1:50 (array tomography)
Goat anti-Mouse IgG3 Alexa Fluor™ 488 Invitrogen Cat# A-21151; RRID: AB_2535784 1:500 (paraffin IHC)
Donkey anti-rabbit Alexa Fluor™ 594 Invitrogen Cat: #A-21207; RRID: AB_141637 1:500 (paraffin IHC)
1:50 (array tomography)
Donkey anti-mouse Alexa Fluor™ 594 Invitrogen Cat: #A-21203; RRID: AB_2535789 1:500 (paraffin IHC)
Donkey anti-goat Alexa Fluor™ 647 Invitrogen Cat:#A-21447; RRID: AB_2535864 1:50 (array tomography)
Goat anti-mouse IgG1 Alexa Fluor™ 594 Invitrogen Cat: #A-21125; RRID: AB_141593 1:50 (array tomography)
Goat anti-mouse IgG1 Alexa Fluor™ 647 Invitrogen Cat: #A21240; RRID: AB_2535809 1:500 (paraffin IHC)
1:50 (array tomography)
Goat anti-rabbit Alexa Fluor™ 647 Invitrogen Cat: #A-21244; RRID: AB_2535812 1:500 (paraffin IHC)
1:50 (array tomography)
Donkey anti-mouse Alexa Fluor® 647 ThermoFisher Cat: #A32787; RRID: AB_2762830 1:50 (array tomography)
Donkey anti-rabbit IRDye 800CW Li-Cor Biosciences Cat:# 925-32213 RRID: AB_2715510 1:5000 (Western blot)
Donkey anti-mouse IRDye 680RD Li-Cor Biosciences Cat# 925-68072 RRID: AB_2814912 1:5000 (Western blot)
Goat anti-rabbit 10nm gold Abcam Cat: #ab39601 RRID:AB_954434 1:25 (immuno-EM)
Goat anti-mouse 10nm gold Abcam Cat: #ab39619 RRID:AB_954440 1:25 (immuno-EM)

Validation

Antibody Validation Publications
Rabbit anti-PSD95 From manufacturer: PSD95 (D27E11) XP® Rabbit mAb detects endogenous levels of total PSD95 protein. Monoclonal antibody is produced by immunizing animals with a synthetic peptide corresponding to residues surrounding Gln53 of human PSD95. Western blots of human brain this antbody recognises a clean band at 95 kDa. Immunofluorescence shows punctate synaptic staining as expected.
We validated using array tomography on PSD95 knockout mouse tissue that there was no staining with this antibody. Colom-Cadena M, Davies C, et al.. Neuron. 2023 Jul;111(14):2170-2183.e6.
Guinea pig anti-PSD95 Manufacturer shows clean 95 kDa band on western blot, synaptic pattern staining on IHC and lack of staining in knockout mouse brain https://sysy.com/product/124308
We in-house validated co-staining between this antibody and the rabbit PSD95 antibody above. Colom-Cadena M, Davies C, et al.. Neuron. 2023 Jul;111(14):2170-2183.e6.
Recombinant Alexa Fluor® 488 Anti-Synaptophysin antibody [YE269] Manufacturer shows:
- Specificity confirmed with SYP knockout cell line validation

- Specificity and sensitivity confirmed in IHC with multi-tissue microarray (TMA) validation
https://www.abcam.com/en-us/products/primary-antibodies/synaptophysin-antibody-ye269-ab32127?srsltid=AfmBOoqjRiMFKC2w2Z6lYveCnwL4ooPpyQ7jSWxjf5sJD18BkJPp-SWJ
We validated in-house by observing co-staining with other synaptophysin antibody staining in human brain with array tomography. Colom-Cadena M, Davies C, et al.. Neuron. 2023 Jul;111(14):2170-2183.e6.
Mouse anti-synaptophysin (SY38) From manufacturer: Abcam's high quality validation processes ensure Anti-Synaptophysin antibody [SY38] (ab8049) has high sensitivity and specificity.
Anti-Synaptophysin antibody [SY38] (ab8049) has been cited over 228 times in peer reviewed journals and is trusted by the scientific community.

We validated in-house by observing co-staining with SV2A staining (another pre-synaptic vesicle protein) in human brain with array tomography. Daniels et al eLife 2023 12:12:e85279.
doi: 10.7554/eLife.85279

Colom-Cadena M, Davies C, et al.. Neuron. 2023 Jul;111(14):2170-2183.e6.
Goat anti-synaptophysin Manufacturer shows expected Western blot and IHC results in human tissue. https://www.rndsystems.com/products/human-synaptophysin-antibody_af5555?gad_source=1&gclid=Cj0KCQjwhr6_BhD4ARIsAH1YdjBzyZgOAn2y2ygXVjIevZofYE9kz_k16WGn0cyKV_k2LKH5o1K5TQsaArJ6EALw_wcB&gclsrc=aw.ds

We validated in-house by observing co-staining with other synaptophysin antibody staining in human brain with array tomography. Giandomeni et al Nat. Neurosci., 2019-03-18;22(4):669-679
Rabbit anti-Synapsin-1 Maufacturer reports Synapsin I (AB1543P) representative staining pattern/morphology in rat hippocampal neuron https://www.merckmillipore.com/AT/en/product/Anti-Synapsin-I-Antibody,MM_NF-AB1543P#overview

We validated by ensuring co-staining with other presynaptic antibodies. Tzioras et al 2023 Cell Rep Med 30;101175
Mouse anti-phosphorylated tau (AT8) Manufacturer reports the antibody was validated by cell treatment to ensure antibody only binds to specific phohspo-tau. They also show staining and western blots with expected pattern and lack of binding to total tau. https://www.thermofisher.com/antibody/product/Phospho-Tau-Ser202-Thr205-Antibody-clone-AT8-Monoclonal/MN1020 Colom-Cadena M, Davies C, et al.. Neuron. 2023 Jul;111(14):2170-2183.e6.
Rabbit anti-oligomeric tau (T22, serum) Collaborator who provided the antibody showed specificity of T22 was examined by ELISA analysis, which shows the specificity of the antibody for tau oligomers (Fig. 1A); T22 does not show any significant reactivity for monomeric tau, Aβ oligomers, Aβ fibrils, α-synuclein oligomers, or α-synuclein fibrils. Lasagna-Reeves et al FASEB J 2012 May;26(5):1946–1959.
Chicken anti-GFAP Manufacturer validated for immunocytochemistry/immunofluorescence (ICC/IF), western blotting (WB), immunohistochemistry (IHC), and immunohistochemistry on frozen sections (IHC-Fr) applications. Cited in over 630 papers. Tzioras et al 2023 Cell Rep Med 30;101175
Rabbit anti-P2RY12 Manufacturer performed orthogonal validation of protein expression using IHC by comparison to RNA-seq data of corresponding target in high and low expression tissues and IHC validation in >40 human samples https://www.atlasantibodies.com/products/primary-antibodies/triple-a-polyclonals/anti-p2ry12-antibody-hpa014518/

We validated co-staining with other microglial antibodies in human brain (CD68 and Iba1). Tzioras et al 2023 Cell Rep Med 30;101175
Mouse anti-CD68 Manufacturer states the antibody labels COS-1 and WOP cells transfected with CD68 cDNA. Unlike other CD68 antibodies, which label both macrophages and myeloid cells, the PG-M1 antibody detects a fixative-resistant epitope on the macrophage-restricted form of the CD68 antigen

We validated co-staining with other microglial antibodies in human brain (P2RY12 and Iba1). Tzioras et al 2023 Cell Rep Med 30;101175
Rabbit anti-GAPDH Manufacturer shows specific western blot and IHC staining https://www.abcam.com/en-us/products/primary-antibodies/gapdh-antibody-loading-control-ab9485
Tzioras et al 2023 Cell Rep Med 30;101175
Mouse anti-synaptogyrin-3 Manufacturer shows detection of Synaptogyrin-3 of mouse, rat and human origin by WB, IP, IF and ELISA. https://www.scbt.com/p/synaptogyrin-3-antibody-e-11?srsltid=AfmBOoq1qB_uMPnZgh5dJbq0ntFWFHT512Y6mS4xHrxm84SIALY31Y83

We validated by ensuring co-staining with other presynaptic antibodies. Zhou et al. Nat Commun. 2017 May 11;8:15295.
Goat anti-human tau Manufacturer shows detection of human Tau in direct ELISAs and Western blots. https://www.rndsystems.com/products/tau-antibody_af3494#product-citations

We validated by co-staining with other tau antibodies. Pickett et al Cell Rep, 2019-12-10;29(11):3592-3604.e5.
Mouse anti-clusterin Manufacturer states validated for use in western blotting (WB), immunoprecipitation (IP), immunofluorescence (IF), immunohistochemistry with paraffin-embedded sections (IHCP), and enzyme-linked immunosorbent assay (ELISA) https://www.scbt.com/p/clusterin-antibody-a-9?gad_source=1&gclid=Cj0KCQjwhr6_BhD4ARIsAH1YdjDkkCd0fEwZ0JY0AmJfpTNQy7Bd6jkSqevh0YyvxwC6Tq-h_vWMNUoaAoznEALw_wcB
Jackson et al. Brain Commun. 2019;1(1):fcz003
Mouse anti-tau 15-25 (Tau13) Manufacturer reports validated for use in western blot and IHC. https://www.biolegend.com/en-gb/products/anti-tau-15-25-antibody-11155?GroupID=GROUP32

We validated by co-staining with other tau antibodies. Pickett et al Cell Rep, 2019-12-10;29(11):3592-3604.e5.
Rabbit anti-histone (H3) Manufacturer states validated for western blot, ICC/IF, and ChIP. Suitable for human and mouse samples. Cited in over 4,700 publications Hesse et al. Acta Neuropathol Commun. 2019;7(1):214.

Plants

Seed stocks	not applicable
Novel plant genotypes	not applicable
Authentication	not applicable
